# Information literacy in the digital age: information sources, evaluation strategies, and perceived teaching competences of pre-service teachers

**DOI:** 10.3389/fpsyg.2024.1336436

**Published:** 2024-03-15

**Authors:** Jessica Trixa, Kai Kaspar

**Affiliations:** Department of Psychology, University of Cologne, Cologne, Germany

**Keywords:** pre-service teachers, information literacy, sources and strategies, perceived teaching competence, learning opportunities, self-efficacy, selective exposure, need for cognition

## Abstract

**Introduction:**

Information literacy has become indispensable in navigating today’s fast-paced media environment, with teachers playing a pivotal role in fostering reflective and critical digital citizenship. Positioned as future gatekeepers, pre-service teachers are the key to teaching media skills and especially information literacy to future generations of pupils. Given the particular challenges facing educators today compared to previous generations, it is important to determine whether the next generation of teachers feel adequately prepared and perceive themselves as competent to pass on these skills to their future pupils. However, previous research has highlighted deficiencies in formal learning opportunities at universities, underscoring the need for further investigation into pre-service teachers’ information acquisition, evaluation practices as well as their perceived relevance to teaching, and person-related factors associated with their perceived competence in teaching information literacy.

**Method:**

An online questionnaire was presented to participants, employing a mixed-method approach. We qualitatively examined the sources of information used by pre-service teachers and the evaluation strategies they employ, while quantitatively analyzing relationships between pre-service teachers’ person-related factors and their perceived teaching competence. Participants assessed their perceived teaching competence, perceived learning opportunities, self-efficacy (general and related to information assessment), perceived informedness, selective exposure, need for cognition, need for cognitive closure, and mistrust in media coverage.

**Results:**

Data from 371 participants revealed digital media dominance in information acquisition over traditional sources, albeit with a prevalence of surface-level evaluation strategies over reflective approaches. Two distinct dimensions of perceived competence in teaching information literacy emerged: one focusing on information assessment while the other centers on the understanding of news creation processes. Perceived competence in teaching information literacy was significantly associated with self-efficacy in information assessment, perceived informedness, selective exposure to information as well as perceived learning opportunities focusing on information evaluation. Moreover, pre-service teachers employing diverse information evaluation strategies demonstrated a heightened sense of perceived competence in teaching information assessment.

**Discussion:**

Our results provide valuable insights into the multifaceted nature of pre-service teachers’ perceived competence in teaching information literacy. Theoretical implications for future research as well as practical implications for teacher education and the structure of future curricula are discussed.

## 1 Introduction

The increasing digitization of all areas of life and the spread of digital media technologies have affected society in many ways. Potentially constant access to information on virtually any topic can be incredibly enriching. However, an effective use of this potential requires a skillful use of the technologies that enable this access in the first place as well as a critical engagement with the information presented and the sources that disseminate it. For example, social media platforms have become a critical information source in today’s information eco-system (Shearer and Mitchell, 2021). What causes concern is that the dynamic nature of social media can also favor the proliferation of mis- and disinformation, including fake news (Valtonen et al., 2019). Hence, if individuals rely on information offered to them on social networks, it is vital that they have the necessary skills to validly assess the (lack of) quality of the content presented to them. An important skill in this regard is information literacy. In dealing with information, an information-literate person is characterized by critical handling, targeted research, selection and evaluation of information and its quality (Association of College and Research Libraries, 2000).

What makes information literacy a key element in educational systems is the fact that the flood of information on the internet not only affects adults. Children and adolescents can potentially access information on virtually any topic within a few clicks as well. Today’s technological possibilities have made media literacy education more important than ever and an urgent need for contemporary media literacy education in school curricula has been expressed by many authors and in different contexts (see, e.g., Pérez Rodríguez et al., 2019; Valtonen et al., 2019; Trixa and Breuer, 2020). Schools undoubtedly have a special responsibility when it comes to preparing students for critical and reflective thinking in a modern digital world, and teachers have a key role to play in providing the necessary skills and competencies. The required competencies comprise technical skills but also an increased awareness of the problems that digitization can bring, as well as a broad understanding of the impact of digitization on human interactions and the way people seek and consume online information (Falloon, 2020). Many of today’s teachers, however, grew up in a transitional era and have varying levels of skill and knowledge in using the technology tools that are available to them (Hatlevik and Hatlevik, 2018).

Educators of today face significantly different challenges compared to previous generations. This raises the fundamental question of whether teachers feel adequately prepared to convey the multifaceted concept of information literacy to their students in today’s ever-evolving digital landscape. This is further complicated by the fact that teachers often perceive themselves as having a lower level of competence in information and communications technology (ICT) and media use compared to the importance they attribute to these competencies in teaching (Gutiérrez-Martín et al., 2022). It is widely recognized that both knowledge and beliefs significantly influence teaching quality and learning outcomes (Baumert et al., 2010; Voss et al., 2013) as well as competence (Rott, 2021). Initial studies have begun to investigate the competencies required by educators to promote students’ information literacy, and there is a need for further exploration into the factors affecting teachers’ proficiency in this area (Siddiq et al., 2016; Wu et al., 2022).

Positioned as the gatekeepers of tomorrow, especially today’s cohort of pre-service teachers plays a pivotal role in transmitting their acquired media habits to future generations. Given the influence of teachers’ beliefs and teaching style on their students’ beliefs (see, e.g., Brownlee and Berthelsen, 2008), additional exploration is necessary to clarify pre-service teachers’ perceptions of their competency in teaching information literacy and the factors that influence these perceptions. The present study aims to help filling this gap by investigating what factors determine whether pre-service teachers feel competent to teach information literacy in the classroom and what importance they attribute to this topic in their teaching through three research questions. First, what sources of information do pre-service teachers draw upon when purposefully seeking information? Second, what strategies do pre-service teachers use to evaluate the accuracy and reliability of information on the internet or to identify incorrect information, and how relevant do pre-service teachers think it is that these strategies are taught to their (future) school students? Third, what person-related factors are related to pre-service teachers’ perceived competence in being able to teach information literacy?

### 1.1 Sources of information

The digitalized nature of people’s everyday lives has led to a change in information behavior over the course of the last few years (Newman et al., 2018; Nielsen et al., 2019). A large proportion of the young population does not feel the need to actively seek for news in order to be well informed. Instead, they prefer to rely on information flowing to them from their personal networks (Gil de Zúñiga et al., 2017; Matsa and Shearer, 2018; Gil de Zúñiga and Diehl, 2019). This shift has led to a growing reliance on the internet and social media for information, posing challenges related to the verification of sources and the quality of the information accessed (Ireton and Posetti, 2018; Kalsnes, 2018; McNeill, 2018; Kaspar and Müller-Jensen, 2021; Zimmermann et al., 2022). The global Covid-19 pandemic sparked an infodemic, a term coined by the World Health Organization (WHO), which resulted in confusion and mistrust among people (United Nations, 2020). While some authors criticize the dissemination platforms (see, e.g., Kalsnes, 2018; Jones-Jang et al., 2019), many others point to the necessity of media and information literacy education that can prepare or train users to better cope with or detect fake news (Mele et al., 2017; Mihailidis and Viotty, 2017; McDougall et al., 2018; Jones-Jang et al., 2019; Clayton et al., 2020).

In recent years, increasing attention has been paid to various types of media-related competence concepts, such as information literacy focusing on identifying, locating, evaluating, and using information in digital environments (Jones-Jang et al., 2019). In analogy to literacy as the ability to know and use the right letters to write our words, information literacy can be seen as the ability to navigate the world of information and make the right choices for our personal and professional information needs (Sanches, 2018). One of the competencies expected from information-literate individuals is to be able to critically evaluate information and the process of information seeking (Australian and New Zealand Institute for Information Literacy and Council of Australian University Librarians, 2004). Particularly against this background, it is of high importance to understand from which sources individuals obtain their information. Not all information that can be found online has been reviewed – let alone didactically prepared – as it is the case for school textbooks. This emphasizes the urgency of possessing skills for critically questioning and evaluating sources and information already at a young age. Pre-service teachers play a vital role in educating future generations and guiding students in seeking and interpreting valid information. In light of these challenges, understanding the information search strategies and sources of information used by pre-service teachers is of utmost importance. Research has emphasized the need to explore the relationship between pre-service teachers’ information search and evaluation literacy, as well as their ICT self-efficacy in teaching, to better support them in developing effective information evaluation strategies (Dahlqvist, 2021; Peciuliauskiene et al., 2022). We asked:

*RQ1*: Which sources of information do pre-service teachers draw upon when purposefully seeking information?

### 1.2 Strategies employed for the evaluation of information

Over the last few years, there has been a growing awareness of the need for teachers and students to acquire essential competencies to keep up with the technological developments and to harness the full potential of digital media for enhancing education. To achieve this, education professionals will need different knowledge and skills than most of them have today (Persson, 2006). Studies on teachers’ information literacy have revealed that insufficient information literacy may have an impact on how they transmit information literacy to learners (Durodolu, 2020). At the European level, these circumstances were taken into account by implementing two initiatives: the Digital Education Action Plan (2021–2027) to support the sustainable and effective adaptation of the EU Member States’ education systems to the digital age (European Commission, Directorate-General for Education, Youth, Sport and Culture, 2023) and the Digital Competence of Educators (DigCompEdu), a common framework of reference for teachers’ digital literacy (Redecker, 2017). Within the DigCompEdu Framework, the initial sub-area embraces digital competencies specific to educators, encompassing skills in information and media literacy, such as developing personalized search strategies and adapting them according to the quality of acquired information. So far, only little is known about which strategies teachers use to obtain information and to assess its quality in a reliable manner. Research findings suggest that pre-service teachers have a mainly technical view of information literacy, leading to overlook mental processes such as the definition of the information need or strategy (Botturi and Beretta, 2022). Other findings underscore the influence of search engine affordances and personal factors on pre-service teachers’ selection and evaluation of information (Zimmermann et al., 2022). Dahlqvist (2021) revealed a fundamental gap in research concerning teacher students’ information-seeking behaviors. Given the multifaceted nature of information literacy and the varying levels of scientific soundness of online information, we aim at gaining a more comprehensive understanding of strategies employed to assess information quality. Hence, we formulated the following research question:

*RQ2*: What strategies and rules do pre-service teachers use to evaluate the accuracy and reliability of online information, and how relevant do they consider the teaching of these strategies and rules to their pupils in the classroom (instructional delivery)?

### 1.3 Person-related factors related to the perceived competence in teaching information literacy

Person-related factors of pre-service teachers have been identified as highly relevant to the intended teaching behavior (Rüth et al., 2022) and the quality of the education process (Aydın et al., 2013). Aydın et al. (2013) demonstrated that person-related factors are significantly associated with perceived teaching competencies.

Previous research has extensively explored (pre-service) teachers’ understanding of and skills in digital literacy (see, e.g., Bates et al., 2017; Ghomi and Redecker, 2019; Lázaro-Cantabrana et al., 2019; Falloon, 2020; Maher, 2020). Teachers in particular have an important double role to play in this regard. They are required to possess these competencies themselves, while facing the task of imparting them to their students. Yet, various authors point out that universities are struggling to adequately prepare future teachers in information literacy skills and knowledge (Carr, 1998; Asselin and Doiron, 2003; Kovalik et al., 2011). This raises the fundamental question of whether pre-service teachers feel adequately prepared and competent to teach information literacy in their classrooms. Research has investigated the factors associated with the competence of in-service teachers to improve students’ information literacy (Wu et al., 2022). Yet, little is known about pre-service teachers’ perceived competence in teaching information literacy, particularly regarding their confidence in effectively delivering the content to students and the factors associated with this perception.

**Figure 1:**
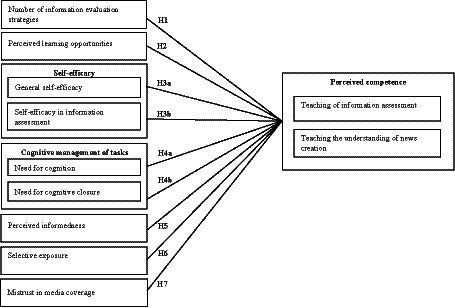
Empirical research model of the present study.

Given the aforementioned dual challenge between pre-service teachers’ own acquisition of competencies and their facilitating role and based on the previous research discussed above, we propose the research model of our study. The model is depicted in Figure 1 and it encompasses different person-related factors that may jointly contribute to an individual’s perceived competence in teaching information literacy. The role of these variables in the illustrated context as well as our hypotheses are presented in the following sections.

#### 1.3.1 Quantity of available information evaluation strategies

One of the biggest barriers teachers face, is a lack of competence and confidence, which often results in their reluctance to incorporate or teach new content. Researchers found that educators who do not feel confident or competent in a particular skill are unlikely to use it (Lin, 2008; Peterson and Palmer, 2011). In order to develop competencies in a specific area, learners must have a profound knowledge and be able to organize this knowledge in a way that makes it easier to retrieve and apply (Bransford et al., 2000). In the context of information seeking, this may suggest that individuals who adopt strategic approaches or have internalized a specific professional rule when seeking information also tend to feel more proficient in addressing information literacy issues overall. Given the lack of research in this particular area, we posed the following exploratory hypothesis:

*H1*: The number of different information evaluation strategies mentioned by pre-service teachers is related to their perceived competence in teaching information literacy.

#### 1.3.2 Perceived learning opportunities

While it is important that everyone acquires information literacy, it might be even more vital for those who plan to become teachers. To facilitate the instruction of these crucial competencies, it is imperative to incorporate suitable (formal) learning opportunities into teacher education (König et al., 2018; Jäger-Biela et al., 2020; Kramer et al., 2020; Hoss et al., 2022). There has been wide recognition that pre-service teachers need to acquire content knowledge about the subjects they will teach as well as the methods and strategies for effectively teaching those subjects (Kovalik et al., 2011). According to Rohlfs (2011), a distinction is made between formal, non-formal and informal learning opportunities. Formal learning opportunities are institutionalized (e.g., in formal educational institutions), follow a structured plan and a formal curriculum with a defined learning objective, and are chronologically graded. Several studies have shown positive effects of learning opportunity characteristics on the acquisition of pedagogical knowledge (e.g., Kunina-Habenicht et al., 2013; Tachtsoglou and König, 2018). In addition, researchers observed that the number of courses attended within the study program was associated with a high increase in knowledge (Watson et al., 2018). Also, positive relations with the amount of learning opportunities were consistently found across countries in international comparison (Blömeke et al., 2010). Based on these findings, it may be expected, that a relation could also be identified with regard to information literacy.

However, Shannon et al. (2019) noted a lack of information literacy training in teacher education programs. Additionally, they observed teachers failing to recognize the concept of information literacy within their teaching curriculum. In Germany, where the present study took place, despite a nationwide commitment to the mandatory implementation of educational objectives regarding digital education (Kultusministerkonferenz, 2016), the consistency of mandatory media literacy courses in the curriculum for teacher training varies across the country. Approximately 66% of higher education institutions require them for the first stage of secondary education, and the percentage drops to only 53% for teacher training aimed at preparing educators for the second stage of secondary education (Monitor Lehrerbildung, 2022). Considering the uncertain availability of specific learning opportunities for information literacy throughout Germany, it is reasonable not to reference the actual learning conditions provided, that is the implemented curriculum (see Robitaille, 1980), but rather focus on students’ perceptions of the learning opportunities they believe they have received in the subject area (attained curriculum). Hence, we formulated the following hypothesis:

*H2*: The perceived formal learning opportunities acquired during the teacher education program are positively related to pre-service teachers’ perceived competence in teaching information literacy.

#### 1.3.3 General self-efficacy and specific self-efficacy in information assessment

Previous research indicates that teachers’ beliefs about their own ability to influence student outcomes can predict student motivation and achievement as well as diverse other facets related to teaching effectiveness (Skaalvik and Skaalvik, 2007). Numerous studies have already dealt with the topic of teaching quality in the past. One factor that has repeatedly shown positive associations in this respect is self-efficacy. Self-efficacy is well-examined in educational research, and its influence on students’ and teachers’ behavior is increasingly recognized (Klassen and Tze, 2014). The concept of self-efficacy derives from Bandura’s social cognitive theory of learning (Bandura, 1997), covers all areas of life and is intended to express an optimistic assessment of general life coping competence. This construct can certainly be described according to its degree of generality or specificity. According to Schwarzer and Jerusalem (2002), domain-specific concepts can be identified, such as teacher self-efficacy expectations. Teacher self-efficacy includes teachers’ beliefs about the ability to successfully cope with difficult demands of their professional lives even under adverse conditions. The authors describe that, *inter alia*, teachers with high self-efficacy create challenging lessons, actively support student learning, and demonstrate patience and attentiveness toward students with learning difficulties. This is due to the fact that they have more confidence in themselves, are more motivated and feel a high level of responsibility for successful and comprehensible lessons (Schwarzer and Jerusalem, 2002). Therefore, it is also conceivable that teachers’ beliefs about being able to assess information reliably and correctly, that is, the domain-specific self-efficacy related to the assessment of information (self-efficacy in information assessment), may also be related to their instructional practices or beliefs regarding teaching information literacy. We hypothesized:

*H3a*: General self-efficacy is positively related to pre-service teachers’ perceived competence in teaching information literacy.

*H3b*: Self-efficacy in information assessment is positively related to pre-service teachers’ perceived competence in teaching information literacy.

#### 1.3.4 Cognitive management of tasks: need for cognition and need for cognitive closure

A construct that is meaningfully different from self-efficacy, but certainly related to it, is the need for cognition (NFC) (Elias and Loomis, 2002). NFC is a person’s propensity to seek out and enjoy activities that require demanding and strained thought and problem-solving (Cacioppo et al., 1996). Overall, the literature seems to suggest that NFC predicts individuals’ interests in a variety of content areas, particularly those requiring deeper or more effortful cognitive processing, such as science or high-level reading (O’Hara et al., 2009; Feist, 2012). Interestingly, especially with regard to information literacy education and the increasing spread of fake news, Haugtvedt and Petty (1992) showed that NFC has also been found to impact the resistance to attitude changes, indicating that altering high-NFC individuals’ evaluations requires a greater amount of information. With regard to the teaching profession, it can be stated that NFC significantly predicts intentions to use ICT in class as well as the individual’s actual ICT behavior (Tanas et al., 2020). Introducing technology in teaching is challenging. Teachers are confronted with the necessity to incorporate novelty and the most recent ideas which at the same time is a complex task that is hard to routinize due to the dynamic nature of software development processes (Tanas et al., 2020). Inferring from these facts, it could be concluded that NFC may also affect the teaching of information literacy. While the content to be covered does not necessarily require the actual use of technology, it does at least require a reflective engagement with it. Since individuals with a high NFC seek out more complex tasks and cognitively demanding activities, it could be assumed that teachers with high NFC find an engagement with a rather demanding and multifaceted topic as information literacy fruitful and also feel competent in facilitating these topics in class. We therefore expected:

*H4a*: NFC is positively related to pre-service teachers’ perceived competence in teaching information literacy.

Another construct associated with cognitive processes that is linked to problem-solving is the need for cognitive closure (NFCC) (DeBacker and Crowson, 2009). NFCC refers to an individual’s desire to simplify complex information and avoid ambiguity (Kruglanski, 1990; Webster and Kruglanski, 1994; Kruglanski and Webster, 1996). Students with higher NFCC, when not under time pressure or other constraints, are generally more inclined to engage in cognitive efforts, such as searching information before making a decision, and conducting additional research before coming to a conclusion (Cará Junior, 2021). Students and educators with a high dispositional NFCC alike may manage knowledge and information intake in ways that lower the likelihood of encountering ambiguity. Teachers with high NFCC may oversimplify course content, leading them to favor assignments that promote only superficial processing of information, such as mere memorization of facts, and consequently do not support learners’ critical thinking (DeBacker and Crowson, 2009). Wronska et al. (2019) found that participants high in NFCC experienced more negative emotions and felt less competent when solving a divergent (vs. convergent) thinking task than those low in NFCC. Transferring these results to the teaching context and more specifically to the instruction of information literacy, it is conceivable that NFCC could also play a role in the perceived competence in teaching an intricate topic such as information literacy in class. Hence, we hypothesized:

*H4b*: NFCC is negatively related to pre-service teachers’ perceived competence in teaching information literacy.

#### 1.3.5 Perceived informedness, selective exposure and mistrust in media coverage

In today’s rich information ecosystem, the ease of publishing allows fake news to rapidly spread, challenging citizens in distinguishing facts from falsehoods (Pennycook and Rand, 2019; Pérez-Escoda et al., 2021). As Metzger et al. (2010) pointed out, seeking the advice of others is a vital strategy to verify, evaluate, and validate online information. People often trust recommendations from friends and family more than other sources. Credibility plays a central role in the persuasiveness of media content (Zimmermann et al., 2022). Within social media contexts, the persuasiveness and perceived usefulness of a message (e.g., Djafarova and Rushworth, 2017; Lou and Yuan, 2019) are determined by factors such as the source’s alleged expertise and trustworthiness, as outlined in the source credibility model (*cf.* Lowry et al., 2013; Zimmermann et al., 2023). It can be concluded that the aforementioned shift in how young people obtain information these days poses a significant challenge in today’s networked world, especially if trusted individuals intentionally or unintentionally disseminate misinformation. Echo chambers (Sunstein, 2007) and filter bubbles fueled by algorithms selecting content to our supposed liking (Pariser, 2011) exacerbate the predicament.

Quite a number of studies suggest that individuals tend to select media content that is consistent with their views (Garrett, 2013; Camaj, 2019). This phenomenon is known as selective exposure, whereby people selectively favor information confirming their attitudes to prevent cognitive dissonance (Festinger, 1957). Hart et al. (2009) concluded that people were, on average, about twice as likely to choose information that supported pre-existing opinions as they were to look at conflicting information. In the information-seeking process, confirmation bias, the inclination to favor information that harmonizes with pre-existing beliefs and reject contradictory information, amplifies vulnerability to falsehoods by uncritically accepting them as facts because they align with pre-existing beliefs (Lord et al., 1979; Knobloch-Westerwick et al., 2015; Soon and Goh, 2019). As information-seeking behavior is an essential part of information literacy, it might also be relevant to the instruction of information literacy. For instance, teachers with a high degree of selective exposure might feel confirmed in their knowledge by the way they obtain information. Feeling well-informed may result in individuals feeling confident in their ability to distinguish fake news from real news and, in turn, to be able to pass that knowledge on to students in the classroom. To our knowledge, these factors (i.e., perceived informedness, selective exposure, and mistrust in media coverage) have not yet been associated with pre-service teachers’ perceived competence in teaching information literacy, hence we formulated the following undirected hypotheses:

*H5*: Perceived informedness is related to pre-service teachers’ perceived competence in teaching information literacy.

*H6*: Selective exposure is related to pre-service teachers’ perceived competence in teaching information literacy.

*H7*: Mistrust in media coverage is related to pre-service teachers’ perceived competence in teaching information literacy.

## 2 Materials and methods

### 2.1 Participants and procedure

The present study strategically targeted pre-service teachers as the research sample to capture insights during a crucial developmental stage of their academic journey, allowing to establish a baseline assessment of their perceived competence in teaching information literacy before they enter the workforce. Pre-service teachers are at a critical juncture where they are acquiring the knowledge and skills that will form the foundation of their teaching practice, ensuring that all respondents have similar levels of practical experience in the field. Our sample includes students who are preparing to become teachers across various types of schools in Germany, ensuring a comprehensive representation of pre-service teachers in the national context. The majority of students indicated German or English as their primary or secondary subject, followed by mathematics. Due to the structure of teacher training in Germany, encompassing different strands of specialization, it is not feasible to isolate one individual subject as major specialization during the course.

The data were collected via an online survey using the software Unipark (Tivian, 2020). Unipark is a widely utilized software for conducting online studies, akin to platforms like Qualtrics (Qualtrics, 2024). Participation is voluntary, and there is no remuneration offered to participants. Additionally, there is no pre-existing pool of individuals who repeatedly participate in studies as Unipark solely offers the platform, with recruitment being self-organized by the researchers. The present non-probability sample (opportunity sample) was recruited via various University distribution lists, social media platforms, including Instagram, X/Twitter, and online groups for pre-service teachers on Facebook in Germany. After clicking on the link to open the study, the participants were informed about the voluntary nature of participation and data protection guidelines. Informed consent to participate in this study was provided by clicking a corresponding box. No sensitive personal data was collected. Participants who ended the survey prematurely were not included in the analyses and all their data were deleted from the dataset.

Overall, 373 pre-service teachers took part in the survey. We initially excluded 2 cases (0.54%) from the analyses due to complete nonresponse or systematic response behavior. Accordingly, the data of 371 participants were included in the analyses. Regarding gender distribution, our sample was composed similarly to the national average in the school year 2022/2023, where female teachers accounted for 73% of the total number of teachers (Statista, 2024b). Our sample included a slightly higher proportion of females, with 299 females, representing 80% of the total sample. The mean age was 25.52 years (*SD* = 6.23); the majority of respondents (69%) were between 21 and 30 years old. The students were either in a bachelor’s or master’s program. At the time of the survey, more than 50% (see Table 1) of the respondents were in the bachelor’s degree stage. The types of schools were not equally distributed in the sample, but all forms were represented. The frequency distribution was equal to that in the population of Germany’s teachers (Statista, 2024a). Gymnasium and comprehensive schools (42.3%) as well as elementary schools (28.8%) made up the largest percentage. In terms of the number of study semesters, there was a wide range in the sample (Min = 1; Max = 20; *M* = 6.27; *SD* = 3.80). A full breakdown of the sample demographics can be found in Table 1.

**Table 1:** Sample demographics.

Variable		*n*	Percentage
Gender	Female	299	80.6
	Male	69	18.6
	Diverse	3	0.8
Age	20 years and younger	55	14.8
	21–30 years	256	69.0
	31–40 years	46	12.4
	41 years and older	14	3.8
Study phase	Bachelor’s degree program	190	51.2
	Master’s degree program	105	28.3
	1st State examination	69	18.6
	2nd State examination	7	1.9
Intended teaching degree	High schools (“Gymnasium”)	157	42.3
	Elementary schools	107	28.8
	Special needs education	44	11.9
	Secondary schools (“Haupt-, Real and Gesamtschule”)	38	10.2
	Vocational schools	25	6.7

### 2.2 Measures

#### 2.2.1 Process of survey development

Different survey instruments were employed in our exploratory study.

First, the sources of information (RQ1) included in the questionnaire were selected to provide a comprehensive understanding of pre-service teachers’ information-seeking behavior across various mediums. We offered respondents a balanced set of options and named the 14 most relevant sources of information as their own possible research sources, including a frequency assessment via rating scales.

Second, participants were asked to name up to five strategies they use to assess the quality of information and to detect misinformation (RQ2). Our goal was to utilize a semi-standardized approach, focusing on open-ended responses, to capture the most significant strategies reflecting participants’ genuine thoughts and experiences, while considering ease of retrieval (*cf.* Hoss et al., 2021). In this context, participants were requested to indicate how relevant they consider the teaching of these strategies and rules to their pupils in the classroom (instructional delivery).

Third, the study used a combination of validated scales and adapted/self-developed scales to test the research model (Figure 1). Concerning our hypotheses (H1 – H7), we aimed to employ established and validated instruments of other sources whenever feasible (e.g., self-efficacy, NFC, and NFCC). When this was not viable, scale content for our target construct was derived either from established definitions of theoretical concepts (e.g., selective exposure) or pertinent previous works (e.g., mistrust in media coverage) to ensure content validity. Subsequently, we consistently assessed both the factorial validity and the internal consistency (Cronbach’s alpha) of the individual factors. A detailed overview of all items of the adapted and self-developed scales used in this study is presented in the Supplementary Table A. For all self-developed indices in this study, we employed exploratory factor analysis to identify underlying structures within the data (see Supplementary Table C) as it aligns with current best practices in ensuring factorial validity. The application of Principal Component Analysis (PCA) or Principal Axis Factoring (PAF) has been vividly discussed in the methodological literature (Thompson and Daniel, 1996). As it is suggested that the practical difference between these two methods regarding interpretation is often negligible (e.g., Thompson, 1992; Onyekachi and Olanrewaju, 2020), the present study compared both methodological perspectives in its factor analyses using varimax rotation. No substantial differences between the two analyses could be noted, as the two accounts suggested the same factorial structure in all present cases. All Kaiser-Meyer-Olkin measures of sampling adequacy were > 0.6, and Bartlett’s tests of Sphericity were significant (*p* < 0.001). Only factors with eigenvalues ≥1 were considered as scales. All constructs used in the study, both the already validated instruments of other sources as well as the self-developed instruments, finally underwent reliability testing via Cronbach’s alpha (values see below).

#### 2.2.2 Sources of information (RQ1)

Items from the study “Jugend, Information, Medien” (Medienpädagogischer Forschungsverbund Südwest, 2019) were adapted to assess which sources of information participants rely on most when searching for information. Respondents were asked to indicate on a scale ranging from 0 (“never”) to 6 (“very often”) how often they used the listed sources of information [e.g., “Search engines (e.g., Google),” “Wikipedia or comparable offers”] when purposefully seeking information. The complete list is presented in the results section (section 3.1).

#### 2.2.3 Employed information evaluation strategies and their relevance for teaching (RQ2)

The instructions for our open-ended questions stated the following: „Not only correct and reliable information can be found on the internet, but also a lot of false and/or misleading information (e.g., fake news). Please indicate up to 5 strategies or rules that you use to evaluate the accuracy and reliability of information on the internet or to identify false information.” In addition, the pre-service teachers were asked: “For each strategy/rule mentioned, please also indicate how relevant you consider the teaching of this strategy/rule to pupils in the classroom,” using a scale ranging from 0 (“not relevant at all”) to 6 (“very relevant”). The strategies were assessed in open statements. These open strategy statements were coded and summarized with the aid of a coding scheme following the standard approach by Mayring (2015), as used in previous research (Kaspar et al., 2010, 2014; Hoss et al., 2021). In a first step, a category system was developed based on the first 10% of the data material by deriving categories from a total of 1,295 statements. This resulted in a category system comprising 17 categories. Subsequently, two persons coded the material independently after prior introduction to the categories. Inter-coder reliability was calculated by Kappa (Cohen, 1960), to ensure the applicability and objectivity of the categories. Agreement was very good across all categories with a minimum κ = 0.922. A consensual agreement was subsequently achieved through discussion in the very few cases of disagreement.

#### 2.2.4 Person-related factors affecting the perceived competence in teaching information literacy (H1–H7)

In the quantitative part of our study, pre-service teachers’ perceived competence in teaching information literacy was our main dependent variable. The number of mentioned strategies, perceived learning opportunities, general self-efficacy and specific self-efficacy related to information assessment, NFC, NFCC, perceived informedness, selective exposure, and mistrust in media coverage were included as independent variables in our model.

As mentioned earlier, exploratory factor analyses (PCA and PAF) were utilized to test the scales’ factorial validity, and internal consistency was calculated as an estimator of reliability. Detailed frequency statistics for all variables are presented in the Supplementary Table B. The operationalization of the dependent and independent variables is described in the following sub-sections.

##### 2.2.4.1 Perceived competence in teaching information literacy

For the assessment of perceived competence in teaching information literacy, we employed items from the 2020 Allensbach study on news literacy in schools (Institut für Demoskopie Allensbach, 2020, K2) and partially adapted the items for the purpose of our study. For 12 items, respondents were asked to indicate on a 7-point scale (0 = “not at all,” 6 = “very much”) how much they felt they were proficient in teaching different facets of information literacy to their students in class. An exploratory factor analysis (PAF) revealed two independent dimensions, one focusing on the assessment of the correctness of information (e.g., “To judge which sources of information can be trusted,” Cronbach’s *α* = 0.86) and one factor addressing the understanding of the creation processes of news (e.g., “To assess the extent to which the media’s own interests influence news production,” *α* = 0.86). One item was removed from the first factor due to low loadings. This analysis resulted in a refinement of our initial research model that instead of one (general) dependent variable we included two (more specific) dependent variables in terms of perceived competence in our analyses: Perceived competence in teaching information literacy with a focus on the assessment of the accuracy of information (shortened as “teaching of information assessment”) and with a focus on the understanding of the creation process of news (shortened as “teaching the understanding of news creation”). Table 2 shows the wordings of all items and the results of the factor analysis.

**Table 2:** Comparison of principal axis factoring (PAF) and principal component analysis (PCA) regarding the dependent variables of the present research model.

	PAF		PCA	
Items	(Rotated) factor loadings		(Rotated) factor loadings	
Perceived competence in teaching the following facets of information literacy:	Information assessment	Understanding of news creation	Information assessment	Understanding of news creation
To recognize false reports or “fake news.”	**0.60**	0.41	**0.66**	0.40
To judge which sources of information can be trusted.	**0.65**	0.33	**0.73**	0.30
To critically scrutinize and evaluate news.	**0.70**	0.37	**0.75**	0.36
To judge whether news is written in a factual or lurid manner.	**0.76**	0.18	**0.83**	0.14
To distinguish news from personal commentary that only reflects the authors’ personal opinion.	**0.64**	0.29	**0.74**	0.25
To assess the extent to which the interests of, e.g., politics, business, or others influence the production of news.	0.29	**0.69**	0.26	**0.76**
To know the political orientation of the various sources of information.	0.30	**0.69**	0.27	**0.75**
To know what different kinds of news sources exist.	0.34	**0.50**	0.33	**0.57**
To understand how news is produced.	0.22	**0.73**	0.18	**0.80**
To assess the extent to which the media’s own interests influence news production.	0.28	**0.68**	0.24	**0.76**
To judge whether news comes from professional journalists or amateurs.	0.41	**0.58**	0.41	**0.63**
Total variance explained	53.76%		61.88%	
KMO	0.900		0.900	
Bartlett’s test	<0.001		<0.001	

Bold values represent the items’ factor allocation.

##### 2.2.4.2 Number of strategies mentioned

The respondents were asked to state up to five strategies they usually employ to evaluate information and their sources (see section 2.2.3). A variable for the number of different strategies mentioned was generated from the coded open statements.

##### 2.2.4.3 Perceived learning opportunities

In order to assess perceived formal learning opportunities, three items reflecting the three usual sequential activities searching/finding (research), analyzing, and evaluating were used in this study. These items were adapted from the Digital Competence Framework for Citizens 2.1 (Carretero et al., 2017) and the Media Competence Framework NRW (Medienberatung, 2019) and were included in the analysis on a single-item basis. Participants were asked to indicate on a scale ranging from 0 (“not at all”) to 6 (“very intensively”) to what extent they felt they had already received learning opportunities on these three main topics as part of their studies (“Research, search, and filter data, information, and digital content,” “Analyze data, information, and digital content as well as their sources,” “Evaluate data, information, and digital content as well as their sources with regard to quality and correctness, including the identification of false information (fake news)”).

##### 2.2.4.4 General self-efficacy and self-efficacy in information assessment

The ASKU (Beierlein et al., 2012) was used to assess general self-efficacy. The scale is an adaptation of the German version of the general self-efficacy expectancy scale by Schwarzer and Jerusalem (1999), and its reliability was evaluated through group studies (between ω = 0.81 to ω = 0.86) (Beierlein et al., 2013). The three items (e.g., “I can usually solve even strenuous and complicated tasks well,” α = 0.83) were measured on a 5-point Likert scale ranging from 1 (“does not apply at all”) to 5 (“totally applies”). Higher scores indicate higher general self-efficacy.

To assess the domain-specific self-efficacy related to information assessment, we adapted the ICT self-efficacy measure from Siddiq et al. (2017). Statements such as “When an assignment/task requires the use of digital tools, I am confident that I will do a great job” of the original scale (α = 0.75) were adapted to fit the information assessment context. The adapted scale comprises three items (e.g., “When I receive conflicting information online, I am confident that I can distinguish misinformation from correct information,” α = 0.80) and uses a 5-point Likert scale ranging from 1 (“does not apply at all”) to 5 (“totally applies”). Higher scores indicate higher self-efficacy in information assessment. The result of the factor analysis supporting a one-dimensional construct and all item wordings and can be found in the Supplementary Table C.

##### 2.2.4.5 Cognitive management of tasks: need for cognition and need for cognitive closure

The measurement of NFC relied on the German adaptation of the NFC scale developed by Bless et al. (1994). It is derived from the original English scale by Cacioppo and Petty (1982), which has undergone validation across various studies (Cacioppo and Petty, 1982; Cacioppo et al., 1983). The German scale consists of 16 items (e.g., “The idea of relying on my thinking ability to accomplish something does not appeal to me“; inverted item), with answer options ranging from −3 (“totally inapplicable”) to +3 (“totally applicable”). Higher scores indicate a higher NFC. The internal consistency of the scale was α = 0.86 in the original German study and α = 0.83 in our study.

The 16-item short scale (16-NCCS) by Schlink and Walther (2007) was employed to assess NFCC. The 16-NCCS is a standardized and cost-effective instrument, consistently demonstrating reliability and high validity in several studies (Schlink and Walther, 2007; Gärtner et al., 2020). It comprises 16 items (e.g., “Any solution to a problem is better than remaining in a state of uncertainty”) with response options ranged from 1 (“do not agree at all”) to 6 (“totally agree”). The internal consistency of the 16-NCCS was α = 0.78 in the original research. In our study, it showed an internal consistency of α = 0.83. Higher scores suggest a higher desire to simplify complex information and avoid ambiguity.

##### 2.2.4.6 Perceived informedness, selective exposure and mistrust in media coverage

For the degree of their own perceived informedness, participants were asked to rate on a single 7-point scale (0 = “not at all,” 6 = “very well”) how well-informed they felt they were about current events and news in the world.

Following previous studies that measured selective exposure indirectly via self-reports (e.g., Kaye et al., 2012; Bardin et al., 2018; Min and Yun, 2018), six items were formulated to assess the individuals’ propensity to consume congruent or affirmative information (e.g., “I do not like consuming media content that presents viewpoints on a topic that I personally tend to disapprove of,” “When consuming media, I consciously make sure that I also deal with points of view that do not coincide with my own,” inverted item, α = 0.71). Respondents were asked to indicate the degree to which each statement applied to them (0 = “does not apply at all,” 6 = “fully applies”). Due to too much similarity with another item, one item was excluded, so that five items were finally used to build a score of selective exposure included in the analyses. The higher the score, the larger the effect of selective exposure, i.e., the tendency to consume information that supports the own opinion. The result of the factor analysis supporting a one-dimensional construct and all item wordings and can be found in the Supplementary Table C.

To assess participants’ mistrust in media coverage, items from the 2020 Allensbach study on news literacy in schools were adapted (Institut für Demoskopie Allensbach, 2020, K2). Participants were asked to indicate their agreement with three items (e.g., “There is less and less emphasis on fact-checking and source-checking in media reports,” α = 0.73) on a response scale ranging from 0 (“do not agree at all”) to 6 (“completely agree”). In this respect, a high score indicates that high mistrust is placed in the media. The result of the factor analysis supporting a one-dimensional construct and all item wordings and can be found in the Supplementary Table C.

## 3 Results

### 3.1 Sources of information (RQ1)

First, we examined which sources of information were most frequently used by pre-service teachers to conduct purposeful information seeking, based on the frequency ratings that ranged between 0 (“never”) to 6 (“very often”). As shown in Table 3, more than 90% of the respondents indicated using search engines (e.g., Google) often (“5” on the employed scale) to very often (“6” on the employed scale) (mean frequency rating was *M* = 5.65, *SD* = 0.79), followed by Wikipedia and comparable offerings (*M* = 3.95, *SD* = 1.62) and scientific literature (*M* = 3.89, *SD* = 1.68). Online news portals of newspapers or magazines (*M* = 3.74, *SD* = 1.63) and video platforms such as YouTube (*M* = 3.63, *SD* = 1.52) were used less frequently but still ranked among the top five sources used for targeted information seeking. There was a visible decrease in usage frequency of the following sources, namely news on television (*M* = 2.27, *SD* = 1.87), online news portals of TV stations (*M* = 1.80, *SD* = 1.79), news on the radio (*M* = 1.74, *SD* = 1.79), podcasts (*M* = 1.71, *SD* = 1.85), news on social media (e.g., Twitter or Facebook) (*M* = 1.68, *SD* = 1.64), printed newspapers (*M* = 1.67, *SD* = 1.66), printed magazines (*M* = 1.56, *SD* = 1.58) and blogs (*M* = 1.16, *SD* = 1.25). These are all sources whose mean rating of usage frequency was below the midpoint of the scale. At the very end of the list was news from email providers (*M* = 0.54, *SD* = 1.00). These results show that there are clear preference media for targeted information seeking and that many sources are used rather marginally or only occasionally. Also, there is considerable variability across the sample, as indicated by the corresponding frequency distributions (see Table 3). The Supplementary Table D additionally provides an overview of how many respondents used how many different sources of information often to very often. About 60% of the respondents (*n* = 224) indicated using at least three or more different sources frequently to very frequently for their information searches.

**Table 3:** Descriptive statistics of the participants’ accessed sources of information.

Information source	*M*	*SD*	Frequency distribution of responses in %						
			0	1	2	3	4	5	6
Search engines (e.g., Google)	5.65	0.79	0.3	0.3	0.3	2.4	4.3	15.1	77.4
Wikipedia or comparable offerings	3.95	1.62	2.2	7.0	10.5	17.8	19.9	21.8	20.8
Scientific journals and reference books	3.89	1.68	2.7	8.9	10.2	15.4	21.3	20.5	21.0
Online news portals of newspapers or magazines	3.74	1.63	5.4	7.0	6.5	20.5	23.7	24.0	12.9
Video platforms (e.g., YouTube)	3.63	1.52	1.9	8.4	12.1	24.0	22.1	19.7	11.9
News on television	2.27	1.87	24.8	16.2	13.7	17.8	12.9	8.9	5.7
Online news portals of TV stations	1.80	1.79	35.6	16.7	13.2	14.0	10.5	7.5	2.4
News on the radio	1.74	1.79	35.0	19.4	14.6	11.9	9.4	5.7	4.0
Podcasts	1.71	1.85	39.4	18.1	9.4	11.6	12.1	5.4	4.0
News on social media (e.g., Twitter or Facebook)	1.68	1.64	31.0	24.3	15.6	12.7	9.7	4.3	2.4
Printed newspapers	1.67	1.66	33.7	21.3	15.6	11.6	10.8	5.4	1.6
Printed magazines	1.56	1.58	34.0	25.9	11.6	14.3	9.4	3.2	1.6
Blogs	1.16	1.25	39.1	26.7	21.0	8.1	3.2	1.1	0.8
News from email providers like gmx, web.de, t-online	0.54	1.00	68.7	18.9	5.9	3.8	2.2	0.3	0.3

7-point scale ranging from 0 (“never”) to 6 (“very often”). The sum of the percentage values might differ from 100% due to rounding.

### 3.2 Information evaluation strategies and their relevance for teaching (RQ2)

We analyzed which strategies/rules pre-service teachers employ to evaluate the reliability of online information and to identify fake news, as well as how relevant the pre-service teachers consider the teaching of each strategy/rule to pupils in the classroom (instructional delivery; 0 = “not relevant at all,” 6 = “very relevant”). In total, the respondents provided 1,295 statements that could be assigned to 16 strategy categories (plus one residual category). As shown in Table 4, the most frequently mentioned strategies were “General source verification” (309) and “Comparison of multiple sources” (248), followed by “Evaluation of scientific nature and trustworthiness of the source” (193) and “Background check of the source (check author, website, imprint)” (165). These strategies are followed in the ranking by “Linguistic style of the source/information (objectivity, neutrality, style)” (71), “Exchange with colleagues/other people” (42), “Presentation style and plausibility of the source/information (spelling mistakes, appearance)” (37), “Logical thinking/prior knowledge” (37), “Relying on sources with subject matter expertise (professional journals, literature)” (36), “Critically questioning hidden interests” (31) and “Using public broadcasting sources (“öffentlich-rechtlicher Rundfunk”)” (24). Only a few statements addressed “social media as an unreliable source” (16) and “source depicts differentiated opinions” (16). The least common strategies named were “Separating facts from opinions” (11), “Reading thoroughly and reflectively, do not skim” (8), and “Checking comments” (6). Although “Separating facts from opinions” was one of the least frequently mentioned strategies, it received the highest relevance rating for instructional delivery (*M* = 5.45, *SD* = 0.69), followed closely by “Using public broadcasting sources” (*M* = 5.38, *SD* = 0.71), which was also only mentioned relatively rarely. In contrast, “General source verification” (*M* = 5.32, *SD* = 0.94) and “Comparison of multiple sources” (*M* = 5.29, *SD* = 0.91) were the two most frequently mentioned strategies and teaching them to students was also rated as very relevant (ranks 3 and 4). “Checking comments” (*M* = 4.00, *SD* = 1.23) received the lowest relevance rating for instructional delivery. However, all strategies were rated at least 4 or higher on the 7-point scale (ranging from 0 to 6), indicating that they were all considered (highly) relevant for instructional delivery.

**Table 4:** Example statements for named strategies for the evaluation of reliability of online information and identification of fake news.

Category of information evaluation strategy	*n*	Mean relevance	Example statements
General source verification	309 (228)	5.32 (0.94)	“Check source citation,” “Check if, how many, and which sources are cited“, “Seek out information about the nature of the source (what are its goals, tendencies?)“, “Use primary/secondary sources?“
Comparison of multiple sources	248 (221)	5.29 (0.91)	“Do not rely on one source, read multiple sources,” “Can the information be found in several independent sources?,” “Majority principle - if the news is reported by different reputable media, it is supposedly safe“
Evaluation of the scientific nature and trustworthiness of the source	193 (166)	5.27 (1.07)	“Verification of the seriousness of the source: can this source provide expert contributions for the subject matter? (existing expertise matching the subject matter)“, “Questioning whether the source of information is reputable: is it a reputable news agency or just tabloid press?,” “Are scientific quality criteria adhered to? Does the article emotionalize or remain factual?“
Background check of the source (check author, website, imprint)	165 (148)	4.92 (1.16)	“Is the author recognizable and what competence does he have? (scientist, journalist, incompetent guy who wants to express his opinion loudly)“, “Background information about the website and the author, check the imprint, name of the website and the like,” “Check imprint: who is responsible for content?”
Linguistic style of the source/information (objectivity, neutrality, style)	71 (65)	4.89 (1.13)	“Generally distrust undifferentiated statements and lurid texts“, “Consider the rhetoric of information,” “Is the tone factual and the text multi-perspective or strongly pushing in one direction (finger-pointing etc.)”
Exchange with colleagues/other people	42 (39)	4.49 (1.35)	“Exchange with friends and acquaintances to get more comparative values“, “Talk to people who may be more familiar with the topic,” “Get opinions from others”
Presentation style and plausibility of the source/information (spelling mistakes, appearance)	37 (36)	4.56 (1.21)	“Check website design/structure“, “Text spelling“, “Presentation of the website (advertising, website has only one article)“
Logical thinking/prior knowledge	37 (34)	4.85 (1.02)	“Use common sense“, “Comparison with my previous knowledge,” “Logical bullshit detector: are the conclusions drawn provable or are they based on plausibility? If so, is this clearly stated?“
Relying on sources with subject matter expertise (professional journals, literature)	36 (34)	4.47 (1.52)	“Use of specialized databases/specialized literature,” “Look up in specialist books,” “Search for specialized literature in the university library“
Critically questioning hidden interests	31 (30)	5.17 (1.05)	“Finding out what possible agenda is behind a piece of information,” “Could the person have a big interest in selling me something? (e.g., Influenced academic of a company selling XY)“, “Question the motivation of the author“
Using public broadcasting sources („öffentlich-rechtlicher Rundfunk“)	24 (24)	5.38 (0.71)	“Researching official sources,” “Institution, e.g., Federal Ministry, Kinderwerk, etc.,” “Use of public service media”
Social media as unreliable source	16 (16)	4.94 (1.44)	“Classic: which site am I on right now? Social media = rarely reliable“,” Only use platforms like Wikipedia to get a general overview and possibly find literature“,” No YouTube“
Source depicts differenciated opinions	16 (16)	4.94 (1.44)	“Different viewpoints,” “Content is illustrated from different dimensions and is not presented as absolute,” “Also deal with the opposing opinion“
Separating facts from opinions	11 (11)	5.45 (0.69)	“Distinguishing opinions from facts, using wording, evidence, etc.“, “Fundamental skepticism about assumptions“, “Fact checker sites (mimika, correctiv, fact checks of the public law)“
Reading thoroughly and reflectively, do not skim	8 (8)	4.63 (1.20)	“Reading news with an open mind, do not be too influenced by your own opinion“, “Reading thoroughly/reading everything (do not skim)“, “Reading the whole text before falling for a headline“
Checking comments	6 (5)	4.00 (1.23)	“Readers’ comments on articles“, “YouTube forums and comments“, “Examine articles for comments“
Other unspecific or statements not fitting any of the categories above	45 (35)	4.26 (1.46)	“The first hits“, “I rarely do, am not an internet freak“, “do not enter personal data”

Column *n* displays the total number of statements and in brackets the number of statements corrected for multiple responses. Relevance ratings ranged from 0 to 6.

### 3.3 Factors related to perceived competence in teaching information literacy (H1 – H7)

We investigated which factors are related to pre-service teachers’ perceived competence in teaching information literacy.

First, we examined the bivariate correlations between the independent variables of the model. As shown in Table 5, these intercorrelations were low in most cases, with only a few exceptions. Participants’ perceived learning opportunities Research and Analyze (*r* = 0.75), Research and Evaluation (*r* = 0.66) and Analyze and Evaluation (*r* = 0.72) were highly positively related. In contrast, all other correlations were small (*r* < |0.30|), except the correlations between NFCC and NFC (*r* = −0.50), general self-efficacy and NFC (*r* = 0.37), self-efficacy in information assessment and perceived informedness (*r* = 0.34), self-efficacy in information assessment and general self-efficacy (*r* = 0.34), and general self-efficacy and NFCC (*r* = −0.30).

**Table 5:** Bivariate correlations between independent variables of the research model (cf. Figure 1).

	*M*	*SD*	1	2	3	4	5	6	7	8	9	10
Independent variable												
Number of information evaluation strategies	3.01	1.25										
Perceived learning opportunity: Research	2.83	1.83	0.05									
Perceived learning opportunity: Analyze	2.54	1.72	0.02	0.75***								
Perceived learning opportunity: Evaluation	1.87	1.65	0.06	0.66***	0.72***							
General self-efficacy	4.00	0.61	−0.03	0.03	0.10*	0.08						
Self-efficacy in information assessment	3.61	0.73	0.05	0.14**	0.16**	0.19***	0.34***					
Need for cognition (NFC)	4.94	0.74	0.10	−0.04	0.01	0.04	0.37***	0.25***				
Need for cognitive closure (NFCC)	3.21	0.69	−0.06	0.05	0.00	−0.04	−0.30***	−0.19	−0.50***			
Perceived informedness	3.68	1.21	−0.07	0.08	0.08	0.06	0.13*	0.34***	0.05	−0.10		
Selective exposure	2.72	0.94	−0.07	0.04	0.06	0.02	−0.08	−0.12*	−0.22***	0.25***	−0.00	
Mistrust in media coverage	2.86	1.27	0.01	−0.05	−0.08	−0.09	−0.02	−0.14**	−0.08	0.16**	−0.17**	0.03

The variable “Perceived learning opportunity” was assessed through three facets, designed to reflect the three sequential activities in information seeking. ***p < 0.001; **p < 0.01; *p < 0.05.

In the next step, we examined the bivariate correlations between independent and dependent variables of the model. We found that all but one independent variable (perceived learning opportunity: research) showed the expected correlation with perceived competence in teaching information assessment. Similarly, all independent variables, except the mentioned number of information evaluation strategies, showed a significant correlation with the perceived competence in teaching the understanding news creation.

In the final step, we computed a multiple regression analysis for each of the two dependent variables to assess the joint contribution of all independent variables and their individual relevance in this context. As shown in Table 6, we found that our research model explained a significant amount of variance in pre-service teachers’ perceived competence in teaching information literacy (teaching of information assessment = 43%, teaching the understanding of news creation = 44%). With respect to the role of individual independent variables in the context of the multiple regression model, we found a nearly identical picture for the two competence dimensions:

The number of information evaluation strategies mentioned (H1) showed a significant positive relation with teaching of information assessment (*β* = 0.10, *p* = 0.029). Regarding teaching the understanding of news creation, no significant relationship was detected.

Among the different facets of perceived learning opportunities (H2), only learning opportunities that addressed the evaluation of data, information, and digital content as well as their sources with regard to quality and correctness (“Evaluation”) showed positive relations to both dependent variables, teaching of information assessment (*β* = 0.16, *p* = 0.007) and teaching the understanding of news creation (*β* = 0.22, *p* = 0.001).

General self-efficacy (H3a) did not show a significant relation to the dependent variables. Self-efficacy in information assessment (H3b), however, had a positive relation to both teaching competences and was the most relevant factor, as indicated by the standardized regression coefficients: teaching of information assessment (*β* = 0.47, *p* < 0.001) and teaching the understanding of news creation (*β* = 0.46, *p* < 0.001).

Regarding the variables related to cognitive task management, neither NFC (H4a) nor NFCC (H4b) showed positive relations to perceived competence in teaching information literacy.

Participants’ perceived informedness (H5) was positively related to both teaching of information assessment (*β* = 0.11, *p* = 0.012) and teaching the understanding of news creation (*β* = 0.15, *p* < 0.001).

In contrast, selective exposure (H6) was negatively related to both teaching of information assessment (*β* = −0.09, *p* = 0.036) and teaching the understanding of news creation (*β* = −0.10, *p* = 0.019).

Finally, mistrust in media coverage did not show a significant relation to any of the dependent variables in the multiple regression model.

### 3.4 Robustness checks

To validate the robustness of our multiple regression results, statistical assumptions of relevance were tested (Poole and O’Farrell, 1971) and, as routinely proposed, bootstrapping (5,000 iterations) was utilized for inferential testing (Hayes and Cai, 2007). The bias-corrected and accelerated bootstrap (BCa) confidence intervals did not include zero for all before reported significant independent variables, so these results can be regarded as robust. To examine the independence of residuals in our regression models, we conducted the Durbin-Watson test (e.g., Dreger et al., 2014), resulting in values between 1 and 2, indicating no detected autocorrelation. In accordance with the collinearity diagnosis criteria set forth by Hair et al. (2013), all variance inflation factor (VIF) values were below 10, and all row-wise variance proportion values were below 0.90, indicating the absence of multicollinearity. Cook’s distance was calculated, revealing no influential outliers, as all cases fell below the threshold of 1 (Cook and Weisberg, 1982).

Beyond our focus on content validity and factorial validity of all measures, some further indications of construct validity can also be inferred from the bivariate correlations observed. In line with theoretical construct definition, we found a negative correlation between NFC and NFCC, and a positive correlation between general self-efficacy and self-efficacy related to information assessment. Moreover, positive correlations between all types of (sequentially related) perceived learning opportunities indicate construct validity.

## 4 Discussion

The present study investigated which sources of information pre-service teachers draw upon and which strategies they use in assessing the reliability of online information as well as the perceived relevance of these strategies for teaching. Further, we explored which factors influence their perceived competence in teaching information literacy in class. The main findings, their implications for research and practice, as well as the limitations will be discussed in the following.

### 4.1 Sources of information (RQ1)

Our findings showed that when pre-service teachers intentionally search for information, “Search engines” are the most commonly utilized source, with “Wikipedia or comparable offerings” and “Scientific journals and reference works” closely following in frequency. “Online news portals of newspapers or magazines” and “Video platforms (e.g., YouTube)” are the subsequent sources in the ranking. In our study, the majority of the respondents (60%) employed at least three different information sources frequently to very frequently in their targeted search for information, suggesting a rather diversified individual information seeking behavior (see Supplementary Table D). The heterogeneity of sources has proven to be beneficial with regard to the identification of fake news in previous studies, as diversifying sources has been shown to reduce susceptibility to fake news (Dubois and Blank, 2018; Sindermann et al., 2021). However, our findings revealed that only some of these sources are subject to standardized and high quality control measures. Sources lacking such professional standards often carry a heightened risk of encountering inaccuracies, often linked to flawed research practices. Intriguingly, none of the traditional media sources – such as television, radio, and newspapers – ranked among the top five information sources. Our findings reflect the fact that, on a national average, media preferences have shifted substantially over the course of one generation (*cf.* Schneller, 2015). This means that the media many of today’s teachers preferably consulted or may still prefer for information purposes have become outdated for the next generation. With regard to the educational landscape, it is of vital importance that teachers become aware of this major change in media consumption, which may continue to undergo further transformation. Likewise, they need to be conscious of the fact that the ranking of young people’s media preferences may differ from the teachers’ own preferences. The prominence of “search engines” as the top choice is not surprising since they serve as gateways to other information sources. However, the use of search engines does not guarantee information quality. Consequently, if the use of search engines is so fundamental to information retrieval, the proper usage of these engines should also form a part of teachers’ media literacy training, as it is more intricate than it appears. An individual’s digital footprint influences future search results, emphasizing the importance of understanding search algorithms and their influence on information presentation. This dynamic can fuel the selective exposure effect, reinforcing echo chambers and filter bubbles. This effect operates at two levels, arising from self-selected content and algorithms that may filter inconsistent information. This is especially significant in light of the fact that future teachers will have the responsibility of teaching young citizens how to properly search for, select, and interpret valid information (Dahlqvist, 2021).

### 4.2 Employed information evaluation strategies and their relevance for teaching (RQ2)

The study’s findings shed light on the prevalent strategies used by pre-service teachers to evaluate information, highlighting both their strengths and potential limitations as well as their perceived relevance for instructional delivery. Our research revealed that the most commonly used strategy among participants was “General source verification,” encompassing reference checking, source quantity and type, and differentiation between primary and secondary sources. This approach could serve as a useful starting point for assessing information quality.

The second-ranked strategy involved comparing multiple sources to verify information across independent and reputable sources. Pursuing a strategy that allows for comparisons is generally beneficial, as it seems to ensure a certain diversity of information. However, this approach may be limited when media sources draw from the same primary sources (e.g., only one press agency or the same author) but frame the information differently. What this strategy certainly can contribute to, however, is to sensitize to the ways in which different narratives are created in the media through different framing of one and the same event.

The third most common category of strategies involves assessing the scientific nature and trustworthiness of the source, often by considering the credibility of the website or author, while the fourth common strategy pertains to evaluating the linguistic style and rhetoric of the information. In contrast, and surprisingly, only few pre-service teachers mentioned relying on logical thinking, prior knowledge, thorough reading, critical questioning, or separating facts from opinions. These important but less frequently mentioned strategies were either taken for granted and therefore not mentioned or, more problematically, perceived as time-consuming and inefficient, with participants favoring more visible and quickly applicable methods, like assessing the publishing source, source citations, and information rhetoric. Yet, it is essential to encourage individuals to engage cognitively with these aspects, especially as false information can appear deceptively realistic. Imparting knowledge, promoting logical thinking, and fostering the ability to distinguish between facts and opinions are critical, necessitating inclusion in pre-service teacher training curricula.

The highest relevance rating for instructional delivery was assigned to the strategy of distinguishing facts from opinions, highlighting its central role in assessing information quality. Similarly important in the form of the relevance rating, “Using public broadcasting services” indicates a commitment to upholding credible media standards. Unsurprisingly, general source checking and comparing multiple sources also ranked high, given their immediate applicability without requiring extensive engagement with the subject matter.

In sum, all mentioned strategies were perceived as (very) relevant, emphasizing the significance of information literacy for school teaching also from the perspective of pre-service teachers. However, most pre-service teachers opted for time-saving and less reflective strategies, prioritizing reputable sources and cross-referencing information. While these widely employed strategies can serve as a solid starting point, relying on them exclusively may lead to misinterpretations because of their superficial nature. In light of the competences required by education policymakers at the European level (European Commission, Directorate-General for Education, Youth, Sport and Culture, 2023), our findings underscore the necessity for pre-service teacher education to prioritize individual cognitive and strategic initiatives, transcending the limitations of superficial comparison strategies. The prevalent approaches are too short-sighted and may not facilitate in-depth, analytical information verification. The present study identified strategies used by pre-service teachers in order to assess the validity of digital content. For practical implications, the effectiveness as well as the efficiency of these strategies still need to be examined in more detail to derive targeted interventions for future teacher education.

### 4.3 The role of person-related factors for perceived competence in the teaching of information literacy

The study aimed to enrich existing literature by offering insights into the psychological and cognitive factors influencing teachers’ perceived competence in teaching information literacy, along with practical implications.

The first important finding is that we identified two independent dimensions of pre-service teachers’ perceived competence in teaching information literacy: teaching of information assessment and teaching the understanding of news creation. At the level of bivariate correlations between person-related factors and these two competence dimensions, we found the assumed significant correlations, with only two exceptions. However, our primary aim was not to obtain bivariate individual correlations but rather to determine the joint contribution of all independent variables to the perception of competence and their individual relative importance. Indeed, the multiple regression analyses revealed that the joint contribution of all independent variables explained a significant proportion of inter-individual variance in pre-service teachers’ perceived competence in teaching information literacy (both competence dimensions). However, the relationships found at the level of bivariate correlations only partially held in the multiple regression. These findings were consistent across both competence dimensions, displaying an almost identical picture and reinforcing the important role of person-related factors in the context of teaching competences.

#### 4.3.1 Number of strategies mentioned (H1)

When individuals employ multiple strategies to evaluate the trustworthiness of information, they are constrained to engage with the content at a deeper level. Beyond examining the source, they cross-reference information and, at best, also critically consider various perspectives. Bransford et al. (2000) emphasized the need for learners to possess a deep understanding of a subject and the ability to skillfully organize their knowledge to develop competencies within that area. In the context of strategies used to verify information, our research showed that students tend to use a strategic approach to evaluate information by using a combination of strategies. We found that the use of several different information evaluation strategies led to an enhanced sense of competence in teaching the assessment of information. The number of strategies mentioned was positively related to this competence, but it was not related to pre-service teachers’ perception that they could teach the processes of news creation to their future students. It is plausible that the content of the strategies the respondents were asked to express, and the competence of teaching information assessment are, by their very nature, more closely related and therefore showed the expected significant relationship. It is also conceivable that teachers’ prior cognitive involvement with this topic at the beginning of the study, specifically in articulating evaluation strategies, subsequently led to an enhanced feeling of competence in this specific area. In general, pre-service teachers who employ a variety of strategies to assess trustworthiness become more adaptable and better prepared to handle new forms of misinformation and disinformation that emerge regularly. In consequence, pre-service teachers do not only enhance their own information literacy, but also develop a deeper understanding, critical thinking skills, and confidence in teaching this vital aspect to their students. This multifaceted approach is crucial in today’s digital age, where the ability to discern reliable information from misinformation is an essential skill. Thus, it could be argued that the employment of multiple strategies to verify the trustworthiness of information increases pre-service teachers’ perceived competence in teaching this essential skill to their future students.

#### 4.3.2 Perceived learning opportunities (H2)

Previous research emphasizes the importance of appropriate perceived (formal) learning opportunities in teacher education (König et al., 2018; Jäger-Biela et al., 2020; Kramer et al., 2020; Hoss et al., 2022). Our findings showed that among the formal learning opportunities in academic education, only those focusing on the evaluation of information showed a significant positive relation to pre-service teacher’s perceived competence in teaching information literacy. As indicated by standardized regression coefficients, this learning opportunity was the second most important variable in explaining perceived competence in teaching both information assessment and news creation processes. This is particularly noteworthy in light of the fact that most respondents reported limited exposure to such training during their studies. On average, respondents indicated a perception of receiving limited learning opportunities regarding the evaluation of information throughout the course of their studies (Supplementary Table B). The finding that perceived learning opportunities focusing on researching and analyzing information were unrelated to perceived competence in teaching information literacy in our multiple regression analyses may seem counterintuitive at first, considering the usual cognitive sequence of these skills from researching to analyzing and finally evaluating. However, the three skills may not be clearly separable, as they are interconnected processes, as also indicated by bivariate correlations. To evaluate information effectively, one must first research and then analyze the gathered content. From the perspective of the study participants, the corresponding learning opportunities in the area of evaluation might have the most direct link to perceived competences in teaching information literacy. Hence, further research and exploration of these three sequential factors may provide additional insights into this relationship. Further, the respondents were asked to indicate their subjective perceptions which can be influenced by various factors, including individual bias and interpretation. Combining these subjective perceptions with an objective assessment of curricula, as exemplarily conducted by Jäger-Biela et al. (2020), could offer a more accurate picture of the formal learning opportunities pre-service teachers encounter at university. Future research could also consider conducting an experiment, manipulating specific learning opportunities to observe their potential causal influence on perceived (or even actual) teaching competence. Overall, our findings suggest ample room for improvement in the provision of topic-relevant learning opportunities to pre-service teachers during their academic education. The respondents’ study programs appear to offer limited opportunities for information literacy education, with a particular need for greater emphasis on the skill of evaluating information, given its vital role in teaching information literacy. Furthermore, adopting a holistic approach that underscores the interconnection between researching, analyzing, and evaluating information may better prepare pre-service teachers for the challenges of the digital information landscape.

#### 4.3.3 General self-efficacy and self-efficacy in information assessment (H3a, H3b)

Our results indicated that self-efficacy related to information assessment exhibited the strongest association (and bivariate correlation) with perceived competence in teaching information literacy for both competence dimensions. In contrast, general self-efficacy did not show a significant association with these competence dimensions. Apparently, teachers who feel highly self-efficacious related to their information assessment behavior feel more confident in handling diverse information and information sources and also perceive themselves as more competent to convey the same skills to their students. The result that domain-specific self-efficacy shows clearer associations with the target constructs has already been shown in other contexts. For example, Kaspar et al. (2023) recently found that especially self-efficacy related to the use of digital media was significantly associated with students’ perceptions of their online learning and their engagement in online courses. Accordingly, universities should prioritize the enhancement of teachers’ self-efficacy in relation to specific teaching activities.

#### 4.3.4 Cognitive management of tasks (H4a, H4b)

NFC and NFCC are constructs used to understand how people approach and engage with cognitive tasks and decision-making processes. Since pre-service teachers have to be able to deal with a wide range of very different and often new information, including new media and cultural artifacts, a connection between NFC/NFCC and their competence of teaching information literacy was expected. The results of our multiple regression analyses, however, show that NFC and NFCC did not significantly contribute to the perceived competence in teaching information literacy. In view of the fact that the expected correlations were found at the bivariate level, it is evident that other factors – such as domain-specific self-efficacy, perceived informedness, and the tendency towards selective exposure – simply play a relatively more important role in the concert of all the variables simultaneously considered in the multiple regression model. From this, a prioritization of target variables could be derived for education, but the result does not mean that NFC and NFCC are fundamentally insignificant for information literacy and its transfer to students.

#### 4.3.5 Perceived informedness (H5), selective exposure (H6), and mistrust in media coverage (H7)

Our findings indicated that the pre-service teachers’ perceived informedness as well as selective exposure were associated with both dimensions of the perceived competence in teaching information literacy. Mistrust in media coverage did not show significant relations with any of the competence dimensions within the multiple regression model.

Perceived informedness was positively related to both teaching of information assessment and teaching the understanding of news creation. We may thus infer that feeling well-informed can enhance pre-service teachers’ confidence in their abilities to teach their students. It is conceivable that teachers who feel well informed are more likely to facilitate informed discussions, address students’ questions, and create an environment where open dialogue and critical thinking are encouraged. Importantly, perceived informedness should correspond to actual valid knowledge, otherwise knowledge illusion can create problems. University education programs can contribute to shaping confident, effective, and responsible educators by integrating real-world examples into their curricula and exposing pre-service teachers to diverse media sources. This approach can help pre-service teachers to bridge abstract concepts with practical situations and prepares them to effectively teach information literacy to the next generation.

Selective exposure was negatively related to both competence dimensions. Our results suggest that pre-service teachers with a high degree of selective exposure might feel less competent in conveying information literacy. Selective exposure is a widespread behavior, and recognizing this common tendency is crucial, as it affects how information is processed and evaluated. Understanding this bias is essential for teaching information literacy effectively, as it allows educators to address potential blind spots, encourage more balanced and inclusive teaching strategies, and promote critical thinking among their students. By acknowledging selective exposure in university education, institutions can better equip pre-service teachers to navigate the challenges posed by biased information consumption and processing. Pre-service teachers could, for instance, be encouraged to reflect on their own information-seeking behaviors and biases and also how these may impact their teaching. Providing resources and training to identify and address selective exposure can foster a balanced and critical approach to information literacy education in future teaching scenarios.

In sum, our findings regarding person-related variables support and expand previous research, suggesting that the combination of high self-efficacy, extensive knowledge, and low confirmation bias reduces susceptibility to fake news (Lord et al., 1979; Knobloch-Westerwick et al., 2015; Soon and Goh, 2019). The research contributes to the existing literature by providing a nuanced understanding of the person-related aspects and their relation to the perceived competence in teaching information literacy, which is essential for informing teacher education and the development of future curricula with respect to information literacy instruction.

### 4.4 General limitations

Some limitations should be considered when interpreting the results. First, in the absence of more thematically pertinent prior research, this study was exploratory in great parts. Consequently, it was not possible to only employ previously established and validated instruments, but it was necessary to develop adapted versions to suit our purposes. As information literacy becomes more and more important not only with respect to todays’ information ecosystem, but also on a societal level, it would be a worthwhile undertaking to systematically develop and validate multi-item scales to assess the specific aspects we examined in our study. The present study could provide a useful initial basis for future projects in this respect. It may also be interesting for future research to compare teachers’ self-assessments of their teaching competence in information literacy with other and more objective measures of their actual information literacy and to examine to what extent they are consistent.

The approach of the present study was distinctly exploratory and purely correlational. Indeed, other variables, such as the field of specialization or the degree of hands-on experience may be relevant as independent variables, especially given that the overall model explains significantly less than 100% of the variance. Additionally, the relationships between dependent and independent variables might be bidirectional and/or additionally affected by further variables. Future research could address the causality question by employing real experiments to observe causal influence on (perceived) teaching competence. However, investigating or demonstrating such causal effects was outside the focus of the present study, the aim of which was to explore potential relations in a first step and thus providing a starting point for future research to advance this heavily under-researched field.

Moreover, the design of our study was cross-sectional and our sample a (voluntary) self-selected convenience sample. The degree of a potential sample bias cannot be finally estimated. In addition, a longitudinal design would provide a fruitful insight into observing the further development over the new teachers’ working years. Although personality traits are not thought to be susceptible for intervention, some of the variables examined here can certainly be changed by teacher training. For example, self-efficacy beliefs can be developed through training or via professional development settings (Klassen and Tze, 2014). If future research can further confirm that teachers’ self-efficacy in information assessment remains crucial in their judgements about their own competence-beliefs, it would be beneficial for academic teacher education programs to provide their students with the kind of support that would lead to the development of strong, resilient self-efficacy beliefs. Additionally, contrasting the perspectives of pre-service teachers with those of experienced educators might offer valuable insights into the continuum of perceived teaching competence across different career stages, providing crucial information for enhancing teacher education programs.

Lastly, all measurements in our study were self-reports. While these are necessary for some constructs (e.g., self-efficacy and NFC), more objective measurements would be desirable. Although a large part of educational research focuses on these individual perceptual aspects, an important next step would be to survey the curriculum content implemented by teachers and actually attained by (university) students.

**Table 6:** Results of the multiple regression analyses testing the research model (H1–H7).

Independent variable	Perceived competence: Teaching of information assessment				Perceived competence: Teaching the understanding of news creation			
	*r*	*B*	β	*p*	*r*	*B*	β	*p*
Number of information evaluation strategies	0.14**	0.08 [0.01, 0.15]	0.10	0.029	−0.02	−0.05 [−0.12, 0.03]	−0.05	0.211
Perceived learning opportunity: Research	0.10	−0.02 [−0.09, 0.05]	−0.04	0.572	0.18***	−0.02 [−0.11, 0.06]	−0.04	0.620
Perceived learning opportunity: Analyze	0.12*	−0.03 [−0.12, −0.05]	−0.06	0.407	0.22***	0.00 [−0.11, 0.13]	0.01	0.962
Perceived learning opportunity: Evaluation	0.21***	0.10 [0.03, 0.16]	0.16	0.007	0.30***	0.15 [0.06, 0.23]	0.22	0.001
General Self-efficacy	0.31***	0.15 [−0.03, 0.31]	0.09	0.092	0.27***	0.05 [−0.12, 0.23]	0.03	0.565
Self-efficacy in information assessment	0.60***	0.65 [0.53, 0.77]	0.47	<0.001	0.59***	0.72 [0.58, 0.85]	0.46	<0.001
Need for cognition (NFC)	0.29***	0.12 [−0.02, 0.27]	0.09	0.079	0.25***	0.13 [−0.04, 0.29]	0.08	0.111
Need for cognitive closure (NFCC)	−0.22***	−0.01 [−0.15, 0.15]	−0.00	0.946	−0.20***	−0.03 [−0.19, 0.14]	−0.02	0.778
Perceived informedness	0.29***	0.09 [0.02, 0.16]	0.11	0.012	0.34***	0.14 [0.06, 0.23]	0.15	<0.001
Selective exposure	−0.19***	−0.10 [−0.20, −0.01]	−0.09	0.036	−0.18***	−0.12 [−0.23, −0.02]	−0.10	0.019
Mistrust in media coverage	−0.13*	−0.02 [−0.09, 0.05]	−0.03	0.547	−0.15**	−0.03 [−0.10, 0.04]	−0.03	0.424
*R*^2^ (adj. *R*^2^)		0.43 (0.41)		<0.001		0.44 (0.42)		<0.001

*r* represents bivariate correlation between independent and dependent variable, *B* values represent unstandardized regression coefficients and their 95%-CI (bias-corrected and accelerated method), *p*-values are based on bootstrapping (5,000 iterations).***p < 0.001; **p < 0.01; *p < 0.05.

## 5 General conclusion

Our exploratory study shed light on critical aspects of information literacy in an era of digital information abundance. Several key findings emerged from our research, providing valuable theoretical insights that can inform future research and practical implications for teacher education and the design of forthcoming curricula pertaining to information literacy instruction. An overview of the main findings and the theoretical and practical implications is presented in Table 7.

**Table 7:** Summary of the key findings and implications of the present study.

Key finding	Theoretical implications	Practical implications
Information retrieval	Imbalance in favor of digital information sources among pre-service teachers, highlighting the prevalent use of search engines as the primary information retrieval tool. Need for theoretical sharpening of the search process in terms of a multi-level process (level 1: search engine; level 2: website/platform; level 3: individual author/program)	Emphasizing the necessity for curricula and training programs that correspond to the evolving requirements in education concerning the transition in information retrieval methods (e.g., search engine proficiency among pre-service teachers, sensitivity to different framings of the same event).
Surface-level information seeking	Tendency towards superficial information-seeking behaviors, underutilizing strategies that involve logical thinking and profound cognitive engagement. Discernment of the relevance of critical evaluation strategies in information assessment.	Recommending the incorporation of dedicated modules into teacher training encouraging cognitive engagement in assessing information, critical thinking and deep, reflective examination of information.
Two dimensions of perceived competence in information literacy education	Identification of two distinct dimensions related to the perceived competence of teaching information literacy: information assessment and understanding of news creation. Information literacy is a multi-facetted construct, whereby profound knowledge on the reception side and the production side are constitutive.	Advocating for curriculum diversification to address the multifaceted aspects of information literacy education. Stressing the need for further research in this regard, also in terms of (pre-service) teachers’ actual competence in teaching these facets.
Dominant role of self-efficacy related to information assessment	Acknowledging the significant impact of domain-specific self-efficacy on the teaching competence of pre-service teachers in information literacy. Conceptual differentiation from other specific forms of domain-specific self-efficacy (e.g., media-related self-efficacy) appears necessary.	Recommendation of integrating training and activities that aim to enhance pre-service teachers’ confidence in their ability to assess information reliably and effectively (e.g., interventions to boost domain-specific self-efficacy through practical experiences and supportive feedback).
Relevance of perceived formal learning opportunities	Highlighting the positive association between perceived formal learning opportunities and perceived teaching competence in the area of information literacy. This underlines the importance of structured, formal learning opportunities, whereby a factual gap between attained (student perspective) and actually implemented (teacher perspective) curriculum content possible.	Advocating for curriculum enhancements that specifically target the three steps: Research, Analyze, and Evaluation. Highlighting the necessity for additional research in this field, particularly considering the content of current curricula.
Role of perceived informedness and selective exposure	Demonstrating the positive and negative associations with teaching competence. Feeling well-informed might enhance pre-service teachers’ confidence in their abilities to teach their students, but knowledge illusion might be a problem; pre-service teachers with a high degree of selective exposure might feel less competent in conveying information literacy, but the direction of effect might be unclear.	Enhancing pre-service teachers’ competence in teaching information literacy through tailored educational programs that integrate real-world examples and diverse media sources. Addressing and mitigating the impact of selective exposure in teaching is crucial, as it enables educators to recognize biased information presentation, to identify potential blind spots, to foster balanced teaching strategies, and to cultivate critical thinking skills.

A notable finding was the shift from traditional media to digital sources for information seeking purposes. Pre-service teachers overwhelmingly reported using search engines as their primary information retrieval tool. This rather superficial information seeking behavior is mirrored by the use of surface-level strategies to assess the reliability of information, over deep, reflective examination. The ability to distinguish factual information from subjective opinions emerged as a critical evaluation strategy for instructional purposes, though it was noticed in only a limited number of students. While this highlights the ubiquity and convenience of search engines for quick access to a multitude of information sources, it also underscores the need for educators to be proficient in using search engines and verification techniques. Additionally, we identified two distinct dimensions within the perceived competence in teaching information literacy, emphasizing the multifaceted character of information literacy education; one dimension is centered on information assessment, while the other is focused on understanding the news creation process. The proposed model showed notable explanatory power and forms a strong foundation for future research. We found that using multiple information evaluation strategies is associated with a higher perception of competence in teaching information assessment. Self-efficacy related to information assessment was identified as the strongest independent variable for both competence dimensions. Our results provide a novel perspective on how domain-specific self-efficacy may impact teaching competence related to information literacy. Furthermore, perceived formal learning opportunities focused on the evaluation of information was positively associated with pre-service teachers’ perceived competence in teaching information literacy. Additionally, feeling well-informed was positively associated with both competence dimensions, while selective exposure showed a negative relation with both competence dimensions. This suggests that educators who feel well-informed and are less prone to selective exposure tendencies may feel better equipped to teach information literacy effectively.

A training program for pre-service teachers could intentionally focus on domain-specific self-efficacy in information assessment by providing opportunities for successful practice, supportive feedback and helping pre-service teachers in coping with distressing emotions that can interfere with effectiveness. In addition, it would be desirable in academic teacher education to raise awareness about cognitive biases, such as the selective exposure effect. Promoting self-reflection on information consumption habits can enhance pre-service teachers’ ability to teach information literacy with sensitivity to the challenges students face in the digital age. Also, integrating dedicated modules on information literacy training covering search engine proficiency, diverse source verification methods, and critical thinking should be considered. This diversification of skills equips future educators to navigate the dynamic information landscape and to effectively impart these skills to their students.

In conclusion, our study advances our comprehension of information literacy among pre-service teachers and underscores the transformative nature of the information landscape. By addressing the practical implications for teacher education and recognizing the need for further research in this regard, we aim to instill a deeper appreciation for the pivotal role information literacy plays in education, especially in the digital age.

## Data availability statement

The raw data supporting the conclusions of this article will be made available by the authors, without undue reservation.

## Ethics statement

Ethical review and approval was not required for the study on human participants in accordance with the local legislation and institutional requirements. Participants who prematurely stopped the survey were not included in the analyses and all of their data were deleted from the dataset. Informed consent to participate in this study was provided by all participants via clicking a corresponding box and all participants voluntarily participated.

## Author contributions

JT: Conceptualization, Data curation, Formal analysis, Investigation, Methodology, Validation, Visualization, Writing – original draft. KK: Conceptualization, Funding acquisition, Investigation, Methodology, Formal analysis, Project administration, Resources, Supervision, Validation, Writing – original draft.

## References

[ref1] Asselin M. Doiron R. (2003). Whither they go: an analysis of the inclusion of school library programs and Services in the Preparation of pre-service teachers in Canadian universities. Behav. Soc. Sci. Libr. 22, 19–32. doi: 10.1300/J103v22n01_03

[ref2] Association of College and Research Libraries (2000). Information literacy competency. Standards for Higher Education. American Library Association. Available at: http://www.ala.org/acrl/standards/informationliteracycompetency

[ref3] Australian and New Zealand Institute for Information Literacy and Council of Australian University Librarians (2004). Australian and New Zealand information literacy framework: Principles, standards and practice (2nd ed). Adelaide: Australian and New Zealand Institute for Information Literacy

[ref4] Aydın M. Bavlı B. Alci B. (2013). Examining the effects of pre-service teachers’ personality traits on their teaching competencies. Int. Online J. Educ. Sci. 5, 575–586.

[ref5] Bandura A. (1997). Self-efficacy: the exercise of control. New York: W.H. Freeman and Company.

[ref6] Bardin B. Vidal P. Facca L. Dumas R. Perrissol S. (2018). The effect of information quality evaluation on selective exposure in informational cognitive dissonance: the role of information novelty. Int. Rev. Soc. Psychol. 31:21. doi: 10.5334/irsp.173

[ref7] Bates D. J. Tiernan D. P. McKeever D. C. (2017). Pre-service teachers’ understanding of information and digital literacy. Ulster University. Available at: https://pure.ulster.ac.uk/ws/portalfiles/portal/75959139/937PRE_SERVICE_TEACHERS_REPORT.pdf

[ref8] Baumert J. Kunter M. Blum W. Brunner M. Voss T. Jordan A.. (2010). Teachers’ mathematical knowledge, cognitive activation in the classroom, and student progress. Am. Educ. Res. J. 47, 133–180. doi: 10.3102/0002831209345157

[ref9] Beierlein C. Kemper C. J. Kovaleva A. Rammstedt B. (2013). Kurzskala zur Erfassung allgemeiner Selbstwirksamkeitserwartungen (ASKU). Methoden Daten Analysen 7, 251–278. doi: 10.12758/mda.2013.014

[ref10] Beierlein C. Kovaleva A. Kemper C. J. Rammstedt B. (2012). Ein Messinstrument zur Erfassung subjektiver Kompetenzerwartungen: Allgemeine Selbstwirksamkeit Kurzskala (ASKU). GESIS-working papers, 2012/17. GESIS - Leibniz-Institut für Sozialwissenschaften

[ref11] Bless H. Wänke M. Bohner G. Fellhauer R. F. Schwarz N. (1994). Need for cognition: Eine Skala zur Erfassung von engagement und Freude bei Denkaufgaben: need for cognition: a scale measuring engagement and happiness in cognitive tasks. Z. Sozialpsychol. 25, 147–154.

[ref12] Blömeke S. Kaiser G. Lehmann R. (2010). TEDS-M 2008. Professionelle Kompetenz und Lerngelegenheiten angehender Mathematiklehrkräfte für die Sekundarstufe I im internationalen Vergleich. Münster: Waxmann Verlag.

[ref13] Botturi L. Beretta C. (2022). Screencasting information literacy. Insights in pre-service teachers’ conception of online search. J. Media Literacy Educ. 14, 94–107. doi: 10.23860/JMLE-2022-14-3-8

[ref14] Bransford J. D. Brown A. L. Cocking R. R. (2000). How people learn. Brain, mind, experience, and school: Expanded edition. Washington, DC: The National Academies Press. doi: 10.17226/9853

[ref15] Brownlee J. Berthelsen D. (2008). “Developing relational epistemology through relational pedagogy” in Knowing, knowledge and beliefs. Epistemological studies across diverse cultures. Ed. Khine M. S. (Dordrecht: Springer), 405–422.

[ref16] Cacioppo J. T. Petty R. E. (1982). The need for cognition. J. Pers. Soc. Psychol. 42, 116–131. doi: 10.1037/0022-3514.42.1.116

[ref17] Cacioppo J. T. Petty R. E. Feinstein J. A. Jarvis W. B. G. (1996). Dispositional differences in cognitive motivation: the life and times of individuals varying in need for cognition. Psychol. Bull. 119, 197–253. doi: 10.1037/0033-2909.119.2.197

[ref18] Cacioppo J. T. Petty R. E. Morris K. J. (1983). Effects of need for cognition on message evaluation, recall and persuasion. J. Pers. Soc. Psychol. 45, 805–818. doi: 10.1037/0022-3514.45.4.805

[ref19] Camaj L. (2019). From selective exposure to selective information processing: a motivated reasoning approach. Media Commun. 7, 8–11. doi: 10.17645/mac.v7i3.2289

[ref20] Cará Junior J. (2021). Cognitive closure as a factor in motivation and perceived learning. In Proceedings of the 4th international conference on research in teaching and education, 18–32.

[ref21] Carr J. A. (1998). Information literacy and teacher education. Available at: https://eric.ed.gov/?id=ED424231

[ref22] Carretero S. Vuorikari R. Punie Y. (2017).DigComp 2.1: The digital competence framework for citizens with eight proficiency levels and examples of use. Luxembourg: Publication Office of the European Union. Available at: https://data.europa.eu/doi/10.2760/38842

[ref23] Clayton K. Blair S. Busam J. A. Forstner S. Glance J. Green G.. (2020). Real solutions for fake news? Measuring the effectiveness of general warnings and fact-check tags in reducing belief in false stories on social media. Polit. Behav. 42, 1073–1095. doi: 10.1007/s11109-019-09533-0

[ref24] Cohen J. (1960). A coefficient of agreement for nominal scales. Educ. Psychol. Meas. 20, 37–46. doi: 10.1177/001316446002000104

[ref25] Cook R. Weisberg S. (1982). Criticism and influence analysis in regression. Sociol. Methodol. 13, 313–361. doi: 10.2307/270724

[ref26] Dahlqvist C. (2021). Information-seeking behaviours of teacher students: a systematic review of quantitative methods literature. Educ. Inf. 37, 259–285. doi: 10.3233/EFI-200400

[ref27] DeBacker T. K. Crowson H. M. (2009). The influence of need for closure on learning and teaching. Educ. Psychol. Rev. 21, 303–323. doi: 10.1007/s10648-009-9111-1

[ref28] Djafarova E. Rushworth C. (2017). Exploring the credibility of online celebrities’ Instagram profiles in influencing the purchase decisions of young female users. Comput. Hum. Behav. 68, 1–7. doi: 10.1016/j.chb.2016.11.009

[ref29] Dreger C. Kosfeld R. Eckey H.-F. (2014). “Ökonometrische Eingleichungsmodelle” in Ökonometrie. Eds. Dreger C. Kosfeld R. Eckey H.-F. (Wiesbaden: Springer Gabler), 19–292.

[ref30] Dubois E. Blank G. (2018). The echo chamber is overstated: the moderating effect of political interest and diverse media. Inf. Commun. Soc. 21, 729–745. doi: 10.1080/1369118X.2018.1428656

[ref31] Durodolu O. O. (2020). Information literacy, self-concept and metacognitive ability of teacher-librarians at the University of Zululand. Libr. Philos. Pract. 1673.

[ref32] Elias S. M. Loomis R. J. (2002). Utilizing need for cognition and perceived self-efficacy to predict academic Performance1. J. Appl. Soc. Psychol. 32, 1687–1702. doi: 10.1111/j.1559-1816.2002.tb02770.x

[ref90] European Commission, Directorate-General for Education, Youth, Sport and Culture, (2023). Digital education action plan 2021–2027: Improving the provision of digital skills in education and training, Publications Office of the European Union. Available at: https://data.europa.eu/doi/10.2766/149764

[ref34] Falloon G. (2020). From digital literacy to digital competence: the teacher digital competency (TDC) framework. Educ. Technol. Res. Dev. 68, 2449–2472. doi: 10.1007/s11423-020-09767-4

[ref35] Feist G. J. (2012). Predicting interest in and attitudes toward science from personality and need for cognition. Personal. Individ. Differ. 52, 771–775. doi: 10.1016/j.paid.2012.01.005

[ref36] Festinger L. (1957). A theory of cognitive dissonance. Stanford, CA: Stanford University Press.

[ref37] Garrett R. K. (2013). Selective exposure: new methods and new directions. Commun. Methods Meas. 7, 247–256. doi: 10.1080/19312458.2013.835796

[ref38] Gärtner J. Bußenius L. Prediger S. Vogel D. Harendza S. (2020). Need for cognitive closure, tolerance for ambiguity, and perfectionism in medical school applicants. BMC Med. Educ. 20:132. doi: 10.1186/s12909-020-02043-2, PMID: 32345278 PMC7189591

[ref39] Ghomi M. Redecker C. (2019). Digital competence of educators (DigCompEdu): development and evaluation of a self-assessment instrument for teachers’ digital competence. In Proceedings of the 11th international conference on computer supported education, 541–548

[ref40] Gil de Zúñiga H. Diehl T. (2019). News finds me perception and democracy: effects on political knowledge, political interest, and voting. New Media Soc. 21, 1253–1271. doi: 10.1177/1461444818817548

[ref41] Gil de Zúñiga H. Weeks B. Ardèvol-Abreu A. (2017). Effects of the news-finds-me perception in communication: social media use implications for news seeking and learning about politics. J. Comput.-Mediat. Commun. 22, 105–123. doi: 10.1111/jcc4.12185

[ref42] Gutiérrez-Martín A. Pinedo-González R. Gil-Puente C. (2022). ICT and media competencies of teachers. Convergence towards an integrated MIL-ICT model. Comunicar 30, 21–33. doi: 10.3916/C70-2022-02

[ref43] Hair J. F. Black W. C. Babin B. J. Anderson R. E. (2013). Multivariate data analysis: advanced diagnostics for multiple regression. Available at: https://mvstats.com/wp-content/uploads/2022/02/Advanced_Regression_Diagnostics.pdf

[ref44] Hart W. Albarracín D. Eagly A. H. Brechan I. Lindberg M. J. Merrill L. (2009). Feeling validated versus being correct: a meta-analysis of selective exposure to information. Psychol. Bull. 135, 555–588. doi: 10.1037/a0015701, PMID: 19586162 PMC4797953

[ref45] Hatlevik I. K. R. Hatlevik O. E. (2018). Examining the relationship between teachers’ ICT self-efficacy for educational purposes, collegial collaboration, lack of facilitation and the use of ICT in teaching practice. Front. Psychol. 9:935. doi: 10.3389/fpsyg.2018.00935, PMID: 29951018 PMC6008425

[ref46] Haugtvedt C. P. Petty R. E. (1992). Personality and persuasion: need for cognition moderates the persistence and resistance of attitude changes. J. Pers. Soc. Psychol. 63, 308–319. doi: 10.1037/0022-3514.63.2.308

[ref47] Hayes A. F. Cai L. (2007). Using heteroskedasticity-consistent standard error estimators in OLS regression: an introduction and software implementation. Behav. Res. Methods 39, 709–722. doi: 10.3758/BF03192961, PMID: 18183883

[ref48] Hoss T. Ancina A. Kaspar K. (2021). Forced remote learning during the COVID-19 pandemic in Germany: a mixed-methods study on students’ positive and negative expectations. Front. Psychol. 12:642616. doi: 10.3389/fpsyg.2021.642616, PMID: 34531779 PMC8439379

[ref49] Hoss T. Ancina A. Kaspar K. (2022). German university students’ perspective on remote learning during the COVID-19 pandemic: a quantitative survey study with implications for future educational interventions. Front. Psychol. 13:734160. doi: 10.3389/fpsyg.2022.734160, PMID: 35282228 PMC8907854

[ref50] Institut für Demoskopie Allensbach (2020). Die Vermittlung von Nachrichtenkompetenz in der Schule. Ergebnisse einer repräsentativen Befragung von Lehrkräften im Februar/März 2020. Available at: https://www.bdzv.de/fileadmin/content/6_Service/6-1_Presse/6-1-2_Pressemitteilungen/2020/Anhaenge/Bericht_Lehrkra__ftebefragung_Nachrichtenkompetenz_neutral.pdf

[ref51] Ireton C. Posetti J. (2018). Journalism, “fake news” and disinformation: a handbook for journalism education and training. Available at: http://unesdoc.unesco.org/images/0026/002655/265552e.pdf

[ref52] Jäger-Biela D. J. Kaspar K. König J. (2020). “Lerngelegenheiten zum Erwerb von digitalisierungsbezogenen Medienkompetenzen: Analysen des Studienangebots und des Nutzungsverhaltens von Lehramtsstudierenden am Fallbeispiel der Universität zu Köln” in Bildung, Schule, Digitalisierung. Eds. Kaspar K. Becker-Mrotzek M. Hofhues S. König J. Schmeinck D. (Münster: Waxmann), 66–72.

[ref53] Jones-Jang S. M. Mortensen T. Liu J. (2019). Does media literacy help identification of fake news? Information literacy helps, but other literacies Don’t. Am. Behav. Sci. 65, 371–388. doi: 10.1177/0002764219869406

[ref54] Kalsnes B. (2018). Fake News. Oxford Research Encyclopedia of Communication. Available at: https://oxfordre.com/communication/view/10.1093/acrefore/9780190228613.001.0001/acrefore-9780190228613-e-809

[ref55] Kaspar K. Burtniak K. Rüth M. (2023). Online learning during the Covid-19 pandemic: how university students’ perceptions, engagement, and performance are related to their personal characteristics. Curr. Psychol., 1–20. doi: 10.1007/s12144-023-04403-9 PMC10025799 37359677

[ref56] Kaspar K. Hamborg K.-C. Sackmann T. Hesselmann J. (2010). Die Effektivität formativer Evaluation bei der Entwicklung gebrauchs- tauglicher Software–eine Fallstudie. Zeitschrift Arbeits Organisationspsychol. 54, 29–38. doi: 10.1026/0932-4089/a000003

[ref57] Kaspar K. König S. Schwandt J. König P. (2014). The experience of new sensorimotor contingencies by sensory augmentation. Conscious. Cogn. 28, 47–63. doi: 10.1016/j.concog.2014.06.006, PMID: 25038534 PMC4154453

[ref58] Kaspar K. Müller-Jensen M. (2021). Information seeking behavior on Facebook: the role of censorship endorsement and personality. Curr. Psychol. 40, 3848–3859. doi: 10.1007/s12144-019-00316-8

[ref59] Kaye B. K. Johnson T. J. Muhlberger P. (2012). Blogs as a source of democratic deliberation. In Dumova T. Fiordo R. (Eds.), Blogging in the global society: Cultural, political and geographical aspects. Hershey: IGI Global.

[ref60] Klassen R. M. Tze V. M. C. (2014). Teachers’ self-efficacy, personality, and teaching effectiveness: a meta-analysis. Educ. Res. Rev. 12, 59–76. doi: 10.1016/j.edurev.2014.06.001

[ref61] Knobloch-Westerwick S. Mothes C. Johnson B. K. Westerwick A. Donsbach W. (2015). Political online information searching in Germany and the United States: confirmation Bias, source credibility, and attitude impacts: political online information search in Germany and United States. J. Commun. 65, 489–511. doi: 10.1111/jcom.12154

[ref62] König J. Doll J. Buchholtz N. Förster S. Kaspar K. Rühl A.-M.. (2018). Pädagogisches Wissen versus fachdidaktisches Wissen? Z. Erzieh. 21, 1–38. doi: 10.1007/s11618-017-0765-z

[ref63] Kovalik C. Jensen M. L. Schloman B. Tipton M. (2011). Information literacy, collaboration, and teacher education. Commun. Inf. Lit. 4, 145–169. doi: 10.15760/comminfolit.2011.4.2.94

[ref64] Kramer C. König J. Strauß S. Kaspar K. (2020). Classroom videos or transcripts? A quasi-experimental study to assess the effects of media-based learning on pre-service teachers’ situation-specific skills of classroom management. Int. J. Educ. Res. 103:101624. doi: 10.1016/j.ijer.2020.101624

[ref65] Kruglanski A. W. (1990). Lay epistemic theory in social-cognitive psychology. Psychol. Inq. 1, 181–197. doi: 10.1207/s15327965pli0103_1

[ref66] Kruglanski A. W. Webster D. M. (1996). Motivated closing of the mind: "seizing" and "freezing.". Psychol. Rev. 103, 263–283. doi: 10.1037/0033-295X.103.2.263, PMID: 8637961

[ref67] Kultusministerkonferenz (2016). Bildung in der digitalen Welt. Strategie der Kultusministerkonferenz. Sekretariat der Kultusministerkonferenz. Available at: https://www.kmk.org/fileadmin/Dateien/veroeffentlichungen_beschluesse/2018/Strategie_Bildung_in_der_digitalen_Welt_idF._vom_07.12.2017.pdf

[ref68] Kunina-Habenicht O. Schulze-Stocker F. Kunter M. Baumert J. Leutner D. Förster D.. (2013). Die Bedeutung der Lerngelegenheiten im Lehramtsstudium und deren individuelle Nutzung für den Aufbau des bildungswissenschaftlichen Wissens. Zeitschrift Pädagogik 59, 1–23. doi: 10.25656/01:11924

[ref69] Lázaro-Cantabrana J. Usart-Rodríguez M. Gisbert-Cervera M. (2019). Assessing teacher digital competence: the construction of an instrument for measuring the knowledge of pre-service teachers. J. New Approaches Educ. Res. 8, 73–78. doi: 10.7821/naer.2019.1.370

[ref70] Lin C.-Y. (2008). A study of pre-service teachers’ attitudes about computers and mathematics teaching: the impact of web-based instruction. Int. J. Technol. Math. Educ. 15, 45–57.

[ref71] Lord C. G. Ross L. Lepper M. R. (1979). Biased assimilation and attitude polarization: the effects of prior theories on subsequently considered evidence. J. Pers. Soc. Psychol. 37, 2098–2109. doi: 10.1037/0022-3514.37.11.2098

[ref72] Lou C. Yuan S. (2019). Influencer marketing: how message value and credibility affect consumer Trust of Branded Content on social media. J. Interact. Advert. 19, 58–73. doi: 10.1080/15252019.2018.1533501

[ref73] Lowry P. B. Wilson D. W. Haig W. L. (2013). A picture is worth a thousand words: source credibility theory applied to logo and website design for heightened credibility and consumer trust. Int. J. Hum. Comput. Interact. 30, 63–93. doi: 10.1080/10447318.2013.839899

[ref74] Maher D. (2020). Pre-service Teachers' digital competencies to support school Students' digital literacies. In Keengwe J. Onchwari G. (Eds.), Handbook of research on literacy and digital technology integration in teacher education. 29–46. Hershey: IGI Global.

[ref75] Matsa K. E. Shearer E. (2018). News use across social media platforms 2018. Pew Research Center. Available at: https://www.pewresearch.org/journalism/2018/09/10/news-use-across-social-media-platforms-2018/

[ref76] Mayring P. (Ed.) (2015). “Qualitative Inhaltsanalyse” in Grundlagen und Techniken. 12th ed (Weinheim: Beltz)

[ref77] McDougall J. Zezulkova M. van Driel B. Sternadel D. (2018). Teaching media literacy in Europe: evidence of effective school practices in primary and secondary education (NESET II report). Publications Office. Available at: https://data.europa.eu/doi/10.2766/613204

[ref78] McNeill L. S. (2018). “My friend posted it and that’s good enough for me!”: source perception in online information sharing. J. Am. Folk. 131, 493–499. doi: 10.5406/jamerfolk.131.522.0493

[ref79] Medienberatung NRW (2019). Broschüre Medienkompetenzrahmen NRW. Medienberatung NRW. Available at: https://medienkompetenzrahmen.nrw/fileadmin/pdf/LVR_ZMB_MKR_Broschuere_2019_06_Final.pdf

[ref80] Medienpädagogischer Forschungsverbund Südwest (2019). JIM-Studie 2019—Jugend, information, Medien. Medienpädagogischer Forschungsverbund Südwest. Available at: https://www.mpfs.de/fileadmin/files/Studien/JIM/2019/JIM_2019.pdf

[ref81] Mele N. Lazer D. Baum M. Grinberg N. Friedland L. Joseph K.. (2017). Combating fake news: An agenda for research and action. Available at: https://shorensteincenter.org/combating-fake-news-agenda-for-research/

[ref82] Metzger M. J. Flanagin A. J. Medders R. B. (2010). Social and heuristic approaches to credibility evaluation online. J. Commun. 60, 413–439. doi: 10.1111/j.1460-2466.2010.01488.x

[ref83] Mihailidis P. Viotty S. (2017). Spreadable spectacle in digital culture: civic expression, fake news, and the role of media literacies in “post-fact” society. Am. Behav. Sci. 61, 441–454. doi: 10.1177/0002764217701217

[ref84] Min H. Yun S. (2018). Selective exposure and political polarization of public opinion on the presidential impeachment in South Korea: Facebook vs. kakaotalk. Korea Observer 49, 137–159. doi: 10.29152/KOIKS.2018.49.1.137

[ref85] Monitor Lehrerbildung (2022). Verpflichtende Angebote zum Erwerb von Medienkompetenz in einer digitalen Welt. Available at: https://www.monitor-lehrerbildung.de/diagramme/verpflichtende-angebote-zum-erwerb-von-medienkompetenz-in-einer-digitalen-welt-absolute-nennungen/?labeltyp=prozent&graustufen=nein

[ref86] Newman N. Fletcher R. Kalogeropoulos A. Levy D. A. L. Nielsen R. K. (2018). Reuters institute digital news report 2018. Available at: https://papers.ssrn.com/sol3/papers.cfm?abstract_id=3245355

[ref87] Nielsen R. K. Newman N. Fletcher R. Kalogeropoulos A. (2019). Reuters institute digital news report 2019. Available at: https://papers.ssrn.com/sol3/papers.cfm?abstract_id=3414941

[ref88] O’Hara R. E. Walter M. I. Christopher A. N. (2009). Need for cognition and conscientiousness as predictors of political interest and voting strategy. J. Appl. Soc. Psychol. 39, 1397–1416. doi: 10.1111/j.1559-1816.2009.00487.x

[ref89] Onyekachi A. M. Olanrewaju S. O. (2020). A comparison of principal component analysis, maximum likelihood and the principal Axis in factor analysis. Am. J. Mathemat. Statistic. 10, 44–54. doi: 10.5923/j.ajms.20201002.03

[ref91] Pariser E. (2011). The filter bubble: What the internet is hiding from you. New York: Penguin Books.

[ref92] Peciuliauskiene P. Tamoliune G. Trepule E. (2022). Exploring the roles of information search and information evaluation literacy and pre-service teachers' ICT self-efficacy in teaching. Int. J. Educ. Technol. High. Educ. 19:33. doi: 10.1186/s41239-022-00339-5, PMID: 35789886 PMC9243709

[ref93] Pennycook G. Rand D. G. (2019). Fighting misinformation on social media using crowdsourced judgments of news source quality. Proc. Natl. Acad. Sci. 116, 2521–2526. doi: 10.1073/pnas.1806781116, PMID: 30692252 PMC6377495

[ref94] Pérez Rodríguez M. A. Delgado-Ponce Á. Mateos P. Romero-Rodriguez L. (2019). Media competence in Spanish secondary school students. Assessing instrumental and critical thinking skills in digital contexts. Educ. Sci. 19, 33–48. doi: 10.12738/estp.2019.3.003

[ref95] Pérez-Escoda A. Pedrero-Esteban L. M. Rubio-Romero J. Jiménez-Narros C. (2021). Fake news reaching young people on social networks: distrust challenging media literacy. Publica 9:24. doi: 10.3390/publications9020024

[ref96] Persson M. (2006). Avision of European teaching and learning: perspectives on the new role of the teacher. European Union. Available at: https://core.ac.uk/download/67039.pdf

[ref97] Peterson S. L. Palmer L. B. (2011). Technology confidence, competence and problem solving strategies: differences within online and face-to-face formats. J. Dist. Educ. 25. Available at: https://www.ijede.ca/index.php/jde/article/download/733/1267?inline=1?inline=1).

[ref98] Poole M. A. O’Farrell P. N. (1971). The assumptions of the linear regression model. Trans. Inst. Br. Geogr. 52, 145–158. doi: 10.2307/621706

[ref99] Qualtrics, (2024). Qualtrics [software]. Available at: https://www.qualtrics.com

[ref100] Redecker C. (2017). European framework for the digital competence of educators: DigCompEdu. European Commission, Joint Research Centre, Publications Office. Available at: https://data.europa.eu/doi/10.2760/159770

[ref101] Robitaille D. F. (1980). Intention, implementation, realisation: the impact of curriculum reform in mathematics. J. Curric. Stud. 12, 299–306. doi: 10.1080/0022027800120403

[ref102] Rohlfs C. (2011). “Ein neuer Bildungsbegriff? Zur Unterscheidung formaler, non-formaler und informeller Bildung: Konturen des aktuellen Bildungsdiskurses” in Bildungseinstellungen. Schule und formale Bildung aus der Perspektive von Schülerinnen und Schülern (Wiesbaden: VS Verlag für Sozialwissenschaften), 33–54.

[ref103] Rott B. (2021). “Epistemological beliefs and critical thinking in pre-service teacher education” in Epistemological beliefs and critical thinking in mathematics. Freiburger Empirische Forschung in der Mathematikdidaktik (Wiesbaden: Springer Spektrum)

[ref104] Rüth M. Birke A. Kaspar K. (2022). Teaching with digital games: how intentions to adopt digital game-based learning are related to personal characteristics of pre-service teachers. Br. J. Educ. Technol. 53, 1412–1429. doi: 10.1111/bjet.13201

[ref105] Sanches T. (2018). “Required skills for teachers: information literacy at the top” in Information literacy in the workplace. Eds. Kurbanoğlu S. Boustany J. Špiranec S. Grassian E. Mizrachi D. Roy L. (Cham: Springer International Publishing), 634–644.

[ref106] Schlink S. Walther E. (2007). Kurz und gut: Eine deutsche Kurzskala zur Erfassung des Bedürfnisses nach kognitiver Geschlossenheit. Z. Sozialpsychol. 38, 153–161. doi: 10.1024/0044-3514.38.3.153

[ref107] Schneller J. (2015). AWA 2015. Auf dem Weg zu neuen Gleichgewichten? Stabilität und Dynamik bei den Mustern der Mediennutzung. Institut für Demoskopie Allensbach. Available at: https://www.ifd-allensbach.de/fileadmin/AWA/AWA_Praesentationen/2015/AWA_2015_Mediennutzung_Schneller.pdf.

[ref9001] Schwarzer R. and Jerusalem M. (1999). Skalen zur Erfassung von Lehrer- und Schülermerkmalen. Dokumentation der psychometrischen Verfahren im Rahmen der Wissenschaftlichen Begleitung des Modellversuchs Selbstwirksame Schulen. Berlin: Freie Universität Berlin.

[ref108] Schwarzer R. Jerusalem M. (2002). Das Konzept der Selbstwirksamkeit. Zeitschrift Pädagogik 44, 28–53. doi: 10.25656/01:7863

[ref109] Shannon C. Reilly J. Bates J. (2019). Teachers and information literacy: understandings and perceptions of the concept. J. Inform. Liter. 13, 41–72. doi: 10.11645/13.2.2642

[ref110] Shearer E. Mitchell A. (2021). News use across social media platforms in 2020. Pew Research Center’s Journalism Project. Available at: https://www.pewresearch.org/journalism/2021/01/12/news-use-across-social-media-platforms-in-2020/

[ref9002] Siddiq F. Hatlevik O. Olsen R. Throndsen I. Scherer R. (2016). Taking a future perspective by learning from the past - A systematic review of assessment instruments that aim to measure primary and secondary school students\u0027 ICT literacy. Educ. Res. Rev. 19, 58–84. doi: 10.1016/j.edurev.2016.05.002

[ref111] Siddiq F. Gochyyev P. Wilson M. (2017). Learning in digital networks – ICT literacy: a novel assessment of students’ 21st century skills. Comput. Educ. 109, 11–37. doi: 10.1016/j.compedu.2017.01.014

[ref112] Sindermann C. Schmitt H. S. Rozgonjuk D. Elhai J. D. Montag C. (2021). The evaluation of fake and true news: on the role of intelligence, personality, interpersonal trust, ideological attitudes, and news consumption. Heliyon 7:e06503. doi: 10.1016/j.heliyon.2021.e06503, PMID: 33869829 PMC8035512

[ref113] Skaalvik E. Skaalvik S. (2007). Dimensions of teacher self-efficacy and relations with strain factors, perceived collective teacher efficacy, and teacher burnout. J. Educ. Psychol. 99, 611–625. doi: 10.1037/0022-0663.99.3.611

[ref114] Soon C. Goh S. (2019). Singaporeans’ susceptibility to false information. Institute of Policy Studies, Lee Kuan Yew School of Public Policy, National University of Singapore.

[ref115] Statista (2024a). Anzahl der Lehrkräfte an allgemeinbildenden Schulen in Deutschland im Schuljahr 2022/2023 nach Schulart [number of teachers at general education schools in Germany in the 2022/2023 school year by type of school]. Available at: https://de.statista.com/statistik/daten/studie/162263/umfrage/anzahl-der-lehrkraefte-nach-schularten/.

[ref116] Statista (2024b). Anzahl der Lehrkräfte an allgemeinbildenden Schulen in Deutschland nach Geschlecht im Schuljahr 2022/2023 [Number of teachers at general education schools in Germany in the 2022/2023 school year by gender]. Available at: https://de.statista.com/statistik/daten/studie/1285371/umfrage/lehrkraefte-in-deutschland-nach-geschlecht/.

[ref117] Sunstein C. R. (2007). Republic.com 2.0. Princeton: Princeton University Press.

[ref118] Tachtsoglou S. König J. (2018). Der Einfluss von Lerngelegenheiten in der Lehrerausbildung auf das pädagogische Wissen angehender Englischlehrkräfte. J. Educ. Res. Online 10, 3–33. doi: 10.25656/01:16131

[ref119] Tanas L. Winkowska-Nowak K. Pobiega K. (2020). The importance of teachers’ need for cognition in their use of Technology in Mathematics Instruction. Front. Psychol. 11:259. doi: 10.3389/fpsyg.2020.00259, PMID: 32153470 PMC7046624

[ref120] Thompson B. (1992). “A partial test distribution for cosines among factors across samples” in Advances in social science methodology. Ed. Thompson B.. 2nd Ed (Greenwich: JAI Press), 81–97.

[ref121] Thompson B. Daniel L. G. (1996). Factor analytic evidence for the construct validity of scores: a historical overview and some guidelines. Educ. Psychol. Meas. 56, 197–208. doi: 10.1177/0013164496056002001

[ref122] Tivian, (2020). Unipark [software]. Available at: https://www.unipark.com/.

[ref123] Trixa J. Breuer J. (2020). Press Start: Digitale Spiele im Unterricht. Grundschule 5, 53–55.

[ref124] United Nations (2020). UN tackles ‘infodemic’ of misinformation and cybercrime in COVID-19 crisis. Available at: https://www.un.org/en/un-coronavirus-communications-team/un-tackling-%E2%80%98infodemic%E2%80%99-misinformation-and-cybercrime-covid-19.

[ref125] Valtonen T. Tedre M. Mäkitalo K. Vartiainen H. (2019). Media literacy education in the age of machine learning. Journal of media literacy. J. Media Literacy Educ. 11, 20–36. doi: 10.23860/JMLE-2019-11-2-2

[ref126] Voss T. Kleickmann T. Kunter M. Hachfeld A. (2013). “Mathematics teachers’ beliefs” in Cognitive activation in the mathematics classroom and professional competence of teachers—Results from the COACTIV project. eds. Kunter M. Baumert J. Blum W. Klusmann U. Krauss S. Neubrand M. (New York: Springer), 249–272.

[ref127] Watson C. Seifert A. Schaper N. (2018). Die Nutzung institutioneller Lerngelegenheiten und die Entwicklung bildungswissenschaftlichen Wissens angehender Lehrkräfte. Z. Erzieh. 21, 565–588. doi: 10.1007/s11618-017-0794-7

[ref128] Webster D. M. Kruglanski A. W. (1994). Individual differences in need for cognitive closure. J. Pers. Soc. Psychol. 67, 1049–1062. doi: 10.1037//0022-3514.67.6.1049 7815301

[ref129] Wronska M. K. Bujacz A. Gocłowska M. A. Rietzschel E. F. Nijstad B. A. (2019). Person-task fit: emotional consequences of performing divergent versus convergent thinking tasks depend on need for cognitive closure. Personal. Individ. Differ. 142, 172–178. doi: 10.1016/j.paid.2018.09.018

[ref130] Wu D. Zhou C. Li Y. Chen M. (2022). Factors associated with teachers’ competence to develop students’ information literacy: a multilevel approach. Comput. Educ. 176:104360. doi: 10.1016/j.compedu.2021.104360

[ref131] Zimmermann M. Engel O. Mayweg-Paus E. (2022). Pre-service teachers’ search strategies when sourcing educational information on the internet. Front. Educ. 7:976346. doi: 10.3389/feduc.2022.976346

[ref132] Zimmermann D. Klee A. Kaspar K. (2023). Political news on Instagram: influencer versus traditional magazine and the role of their expertise in consumers’ credibility perceptions and news engagement. Front. Psychol. 14:1257994. doi: 10.3389/fpsyg.2023.1257994, PMID: 38192389 PMC10773820

[ref133] Zimmermann D. Noll C. Gräßer L. Hugger K.-U. Braun L. M. Nowak T.. (2022). Influencers on YouTube: a quantitative study on young people’s use and perception of videos about political and societal topics. Curr. Psychol. 41, 6808–6824. doi: 10.1007/s12144-020-01164-7

