# Supplementary material for: Information literacy in the digital age: information sources, evaluation strategies, and perceived teaching competences of pre-service teachers
Source: Front Psychol. 2024 Mar 15;15:1336436. doi: 10.3389/fpsyg.2024.1336436 (PMC10978670; doi:10.3389/fpsyg.2024.1336436)
Supplement: Supplementary file 1 [file Table_1.PDF]

Online Supplementary File

1 Supplementary Tables

1.1 Supplementary Table A.

Item wording of the German questionnaire used in data collection and English translation.

Variable	German item	English item
Self-efficacy in information assessment (adapted from ICT self-efficacy by Siddiq et al., 2017)	1. Ich bin mir sicher, dass ich die Glaubwürdigkeit und Qualität von Nachrichten aus Online-Quellen korrekt einschätzen kann.<br>2. Wenn ich online widersprüchliche Informationen präsentiert bekomme, bin ich zuversichtlich, dass ich Falschinformationen von korrekten Informationen unterscheiden kann.<br>3. Ich bin mir sicher, dass ich die wahren Hintergründe und Absichten von Informationen im Internet einordnen und bewerten kann.	1. I am confident that I can correctly assess the credibility and quality of news from online sources.<br>2. When I receive conflicting information online, I am confident that I can distinguish misinformation from correct information.<br>3. I am confident that I can contextualize and evaluate the true background and intentions of information on the Internet.
Perceived learning opportunities (adapted from the Digital Competence Framework for Citizens 2.1 and the Media Competence Framework NRW (Medienkompetenzrahmen NRW)	Wir würden gerne von Ihnen wissen, inwieweit in den von Ihnen im Studium bisher besuchten Lehrveranstaltungen folgende Inhalte bzw. Themen behandelt wurden. Nutzen Sie für Ihre Einschätzung bitte die vorgegebene Skala von 0 (= gar nicht) bis 6 (= sehr intensiv).<br>• Recherchieren, suchen und filtern von Daten, Informationen und digitalen Inhalten.<br>• Analysieren von Daten, Informationen und digitalen Inhalten sowie deren Quellen.<br>• Bewertung von Daten, Informationen und digitalen Inhalten sowie deren Quellen bezüglich Qualität und Korrektheit, einschließlich der Identifikation von Falschmeldungen (Fake News).	We would like to know to what extent the following content or topics have been covered in the courses you have attended so far. Please use the given scale from 0 (= not at all) to 6 (= very intensively) for your assessment.<br>• Research, search, and filter data, information, and digital content.<br>• Analyze data, information, and digital content as well as their sources.<br>• Evaluate data, information, and digital content as well as their sources with regard to quality and correctness, including the identification of false information (fake news).

Note. The items used in the questionnaire were in German language. The respective English translations are only provided for international readability. Self-efficacy in information assessment was measured on a scale ranging from 1 (“does not apply at all”) to 5 (“totally applies”). Perceived learning opportunities were measured on a scale ranging from 0 (“not at all”) to 6 (“very intensively”).

Supplementary Table A (continued)

Variable	German item	English item
Perceived informedness	Selbstwahrgenommene Informiertheit<br><br>Einmal ganz allgemein gefragt: Wie gut fühlen Sie sich über das aktuelle Geschehen informiert, also darüber, was gerade in Deutschland und der Welt los ist? Sie können Ihre Meinung auf der Skala von 0 (= gar nicht gut) bis 6 (= sehr gut) angeben.	Perceived informedness<br><br>In general terms, how well informed do you feel about current events, i.e. what's going on in Germany and the world right now? You can state your opinion on the scale from 0 (= not at all) to 6 (= very well).
Selective exposure<br>(Self-developed scale)	Im Alltag ist die Zeit oft knapp und die Fülle an Informationen groß. Wie gehen Sie daher bei Ihrer Suche und Auswahl von Medieninhalten vor? Bitte geben Sie für jede der folgenden Aussagen an, wie sehr diese auf Sie persönlich zutrifft. Nutzen Sie dazu bitte die Skala von 0 (= trifft gar nicht zu) bis 6 (= trifft voll und ganz zu).<br><br>• Wann immer es geht, konsumiere ich Medieninhalte, die mit meiner Denkweise und meinen Einstellungen grundsätzlich zusammenpassen.<br>• Ich konsumiere ungern Medieninhalte, in denen Betrachtungsweisen auf ein Thema präsentiert werden, die ich persönlich eher ablehne.<br>• Ich konsumiere bevorzugt Medieninhalte, die meine persönlichen Entscheidungen und Verhaltensweisen unterstützen und nicht in Frage stellen.<br>• Ich konsumiere gerne auch Medieninhalte, in denen Einstellungen und Meinungen vertreten werden, die nicht mit meinen eigenen übereinstimmen.<br>• Ich achte beim Medienkonsum bewusst darauf, mich auch mit Sichtweisen zu befassen, die nicht meiner eigenen entsprechen.	In everyday life, time is often short and there is a vast abundance of information. What do you therefore do when searching for and selecting of media content? For each of the following statements, please indicate how much it applies to you personally. Please use the scale from 0 (= does not apply at all) to 6 (= fully applies).<br><br>• Whenever possible, I consume media content that is essentially consistent with my way of thinking and my attitudes.<br>• I don’t like consuming media content that presents viewpoints on a topic that I personally tend to disapprove of.<br>• I prefer to consume media content that supports rather than challenges my personal choices and behaviors.<br>• I also like to consume media content that expresses attitudes and opinions that do not match my own.<br>• When consuming media, I consciously make sure that I also deal with points of view that do not coincide with my own.

Note. The items used in the questionnaire were in German language. The respective English translations are only provided for international readability. Perceived informedness was measured on a scale from 0 (“not at all”) to 6 (“very well”). Selective exposure was measured on a scale from 0 (“does not apply at all”) to 6 (“fully applies”).

Supplementary Table A (continued)

Variable	German item	English item
Mistrust in media coverage (Adapted from IfDA, 2020)	Nachfolgend finden Sie drei Aussagen über die Medienberichterstattung in Deutschland. Inwieweit stimmen Sie den folgenden Aussagen zu? Bitte geben Sie Ihre Meinung auf der Skala von 0 (= stimme gar nicht zu) bis 6 (= stimme voll und ganz zu) an.<br>• Es wird in den Medien immer weniger Wert auf eine Prüfung von Fakten und Quellen gelegt.<br>• In den Medien werden immer mehr falsche Nachrichten, sogenannte 'Fake News', verbreitet.<br>• Die Qualität der Medienberichterstattung wird immer schlechter.	Below you can find three statements about media coverage in Germany. To what extent do you agree with the following statements? Please indicate your opinion on a scale from 0 (= don't agree at all) to 6 (= completely agree).<br>• There is less and less emphasis on fact-checking and source-checking in media reports.<br>• More and more false information, so-called "fake news," is being disseminated in the media.<br>• The quality of media coverage is getting worse and worse.

Note. The items used in the questionnaire were in German language. The respective English translations are only provided for international readability. Mistrust in media coverage was measured on a scale from 0 ("don't agree at all") to 6 ("completely agree").

Supplementary Table A (continued)

Variable	German item	English item
Perceived competence in teaching information literacy<br><br>(Adapted fom IfDA, 2020)	Wahrgenommene Lehrkompetenz von Informationskompetenz<br><br>Stellen Sie sich vor, Sie müssten morgen einen eigenen Unterricht durchführen. Wie sehr fühlen Sie sich aktuell in der Lage, die folgenden Kompetenzen angemessen an Schülerinnen und Schüler vermitteln zu können? Bitte nutzen Sie dazu die Skala von 0 (= gar nicht) bis 6 (= sehr).<br><br>• Falschmeldungen bzw. „Fake News“ erkennen können.<br>• Einschätzen können, welchen Informationsquellen man vertrauen kann.<br>• Nachrichten kritisch hinterfragen und einordnen können.<br>Beurteilen können, ob Nachrichten sachlich oder reißerisch geschrieben sind.<br>• Nachrichten von persönlichen Kommentaren, die ausschließlich die persönliche Meinung der Autor*innen widerspiegelt, trennen können.<br>• Nachrichten verstehen und korrekt wiedergeben können.<br>• Einschätzen können, wieweit die Interessen z. B. von Politik, Wirtschaft oder anderen die Entstehung von Nachrichten beeinflussen.<br>• Die politische Ausrichtung verschiedener Informationsquellen kennen.<br>• Wissen, was für unterschiedliche Nachrichtenquellen es gibt.<br>• Verstehen, wie Nachrichten entstehen.<br>• Einschätzen können, wieweit eigene Interessen der Medien die Entstehung von Nachrichten beeinflussen.<br>• Beurteilen können, ob Nachrichten von professionellen Journalistinnen und Journalisten oder von Laien stammen.	Perceived competence in teaching information literacy<br><br>Imagine you had to teach your own class tomorrow. How much do you currently feel you are able to adequately teach the following competencies to students? Please use the scale from 0 (= not at all) to 6 (= very much).<br><br>• To recognize false reports or "fake news".<br>• To judge which sources of information can be trusted.<br>• To critically scrutinize and evaluate news.<br>• To judge whether news is written in a factual or lurid manner.<br>• To distinguish news from personal commentary that only reflects the authors' personal opinion.<br>• To understand and correctly reproduce news.<br>• To assess the extent to which the interests of, e.g., politics, business, or others influence the production of news.<br>• To know the political orientation of the various sources of information.<br>• To know what different kinds of news sources exist.<br>• To understand how news is produced.<br>• To assess the extent to which the media's own interests influence the creation of news.<br>• To judge whether news comes from professional journalists or amateurs.

Note. The items used in the questionnaire were in German language. The respective English translations are only provided for international readability. Perceived competence in teaching information literacy was measured on a scale from 0 (“not at all”) to 6 (“very much”).

Supplementary Table A (continued)

Variable	German item	English item
Source of information<br><br>(Adapted from JIM Studie, 2019)	Auf welche Informationsquellen greifen Sie zurück, wenn Sie zielgerichtet nach Informationen suchen? Bitte geben Sie an, wie häufig Sie die folgenden Informationsquellen nutzen und verwenden Sie dazu die Skala von 0 (= nie) bis 6 (= sehr häufig).<br><br>• Suchmaschinen (z. B. Google)<br>• Videoplattformen (z. B. YouTube)<br>• Wikipedia oder vergleichbare Angebote<br>• Nachrichten in Social Media (z. B. Twitter oder Facebook)<br>• Blogs<br>• Online-Nachrichtenportale von Zeitungen oder Zeitschriften<br>• Nachrichten bei Providern wie gmx, web.de, t-online<br>• Online-Nachrichtenportale von TV-Sendern<br>• Gedruckte Zeitungen<br>• Gedruckte Zeitschriften<br>• Wissenschaftliche Fachzeitschriften und Fachbücher<br>• Nachrichten im Fernsehen<br>• Nachrichten im Radio<br>• Podcasts	Which sources of information do you rely on when you search for information in a targeted manner? Please indicate how often you use the following sources of information, using the scale from 0 (= never) to 6 (= very often).<br><br>• Search engines (e.g., Google)<br>• Video platforms (e.g., YouTube)<br>• Wikipedia or comparable offers<br>• News in social media (e.g., Twitter or Facebook)<br>• blogs<br>• Online news portals of newspapers or magazines<br>• News from providers such as gmx, web.de, t-online<br>• Online news portals of TV stations<br>• Printed newspapers<br>• Printed magazines<br>• Scientific journals and reference books<br>• News on television<br>• News on the radio<br>• Podcasts

Note. The items used in the questionnaire were in German language. The respective English translations are only provided for international readability. Source of information was measured on a scale ranging from 0 (“never”) to 6 (“very often”), numeric markers from 0 to 6 but only verbal markers at the end poles of the scale.

1.2 Supplementary Table B.

Descriptive statistics of all variables in the empirical research model.

Variable	*M*	*SD*	Frequency in %						
			0	1	2	3	4	5	6
Perceived learning opportunity: Research	2.83	1.83	13.2	14.3	17.8	15.4	17.0	15.4	7.0
Perceived learning opportunity: Analyze	2.54	1.72	14.8	15.6	20.2	19.1	15.4	9.7	5.1
Perceived learning opportunity: Evaluation	1.87	1.65	26.1	21.6	20.8	12.9	10.0	6.2	2.4

Note. n = 371; 7-point scale ranging from 0 (“not at all”) to 6 (“very intensively”).

Variable	*M*	*SD*	Frequency in %				
			1	2	3	4	5
General self-efficacy	4.00	0.61	-	1.6	15.9	60.1	22.4
Self-efficacy in information assessment	3.61	0.73	0.8	6.2	33.7	49.1	10.2

Note. n = 371; 5-point scale ranging from 1 (“does not apply at all”) to 5 (“totally applies”).

Variable	*M*	*SD*	Frequency in %						
			–3	–2	–1	0	+1	+2	+3
Need for cognition (NFC)	4.94	0.74	-	-	2,7	21.3	50.4	24.0	1.6

Note. n = 371; 7-point scale ranging from –3 (“totally inapplicable”) to +3 (“totally applicable”).

Supplementary Table B (continued)

Variable	*M*	*SD*	Frequency in %					
			1	2	3	4	5	6
Need for cognitive closure (NFCC)	3.21	0.69	0.3	13.7	52.6	29.4	4.0	-

Note. *n* = 371; 6-point scale ranging from 1 (“don’t agree at all) to 6 (“totally agree”).

Variable	*M*	*SD*	Frequency in %						
			0	1	2	3	4	5	6
Perceived informedness	3.68	1.21	1.1	2.4	13.7	23.7	33.2	21.3	4.6

Note. *n* = 371; 7-point scale ranging from 0 (“not at all”) to 6 (“very well”).

Variable	*M*	*SD*	Frequency in %						
			0	1	2	3	4	5	6
Selective exposure	2.72	0.94	0.5	1.6	11.6	34.8	34.0	15.1	-

Note. *n* = 371; 7-point scale ranging from 0 (“does not apply at all”) to 6 (“fully applies”).

Variable	*M*	*SD*	Frequency in %						
			0	1	2	3	4	5	6
Mistrust in media coverage	2.86	1.27	2.7	12.7	24.3	29.6	19.9	8.0	2.1

Note. *n* = 371; 7-point scale ranging from 0 (“don’t agree at all”) to 6 = (“completely agree”).

Supplementary Table B (continued)

Variable	*M*	*SD*	Frequency in %						
			0	1	2	3	4	5	6
Perceived competence: Teaching of information assessment	4.31	3.63	-	1.1	5.4	11.1	36.4	36.1	10.0

Note. n = 371; 7-point scale ranging from 0 (“not at all”) to 6 (“very much”).

Variable	*M*	*SD*	Frequency in %						
			0	1	2	3	4	5	6
Perceived competence: Teaching the understanding of news creation	3.63	1.41	0.3	2.7	12.7	25.3	32.9	20.8	5.4

Note. n = 371; 7-point scale ranging from 0 (“not at all”) to 6 (“very much”).

1.3 Supplementary Table C.

Comparison of Principal Axis Factoring (PAF) and Principal Component Analysis (PCA) including factor loadings for all self-developed scales used in the present study.

	PAF		PCA	
Items	(Rotated) Factor loadings		(Rotated) Factor loadings	
Perceived competence in teaching the following facets of information literacy:	Information assessment	Understanding of news creation	Information assessment	Understanding of news creation
1. To recognize false reports or "fake news".	**.60**	.41	**.66**	.40
2. To judge which sources of information can be trusted.	**.65**	.33	**.73**	.30
3. To critically scrutinize and evaluate news.	**.70**	.37	**.75**	.36
4. To judge whether news is written in a factual or lurid manner.	**.76**	.18	**.83**	.14
5. To distinguish news from personal commentary that only reflects the authors' personal opinion.	**.64**	.29	**.74**	.25
6. To assess the extent to which the interests of, e.g., politics, business, or others influence the production of news.	.29	**.69**	.26	**.76**
7. To know the political orientation of the various sources of information.	.30	**.69**	.27	**.75**
8. To know what different kinds of news sources exist.	.34	**.50**	.33	**.57**
9. To understand how news is produced.	.22	**.73**	.18	**.80**
10. To assess the extent to which the media's own interests influence news production.	.28	**.68**	.24	**.76**
11. To judge whether news comes from professional journalists or amateurs.	.41	**.58**	.41	**.63**
KMO	.900		.900	
Bartlett's Test	<.001		<.001	

Note. Bold values represent the factor allocations.

Supplementary Table C (continued)

	PAF	PCA
Items	Factor loadings	Factor loadings
Selective exposure		
1. Whenever possible, I consume media content that is essentially consistent with my way of thinking and my attitudes.	.49	.61
2. I don’t like consuming media content that presents viewpoints on a topic that I personally tend to disapprove of.	.58	.69
3. I prefer to consume media content that supports rather than challenges my personal choices and behaviors.	.63	.72
4. I also like to consume media content that expresses attitudes and opinions that do not match my own.	.67	.74
5. When consuming media, I consciously make sure that I also deal with points of view that do not coincide with my own.	.52	.63
KMO	.711	.711
Bartlett’s Test	<.001	<.001

Note. Only one component was extracted. The solution could therefore not be rotated.

Supplementary Table C (continued)

Items	PAF<br>Factor loadings	PCA<br>Factor loadings
Self-efficacy in information assessment		
1. I am confident that I can correctly assess the credibility and quality of news from online sources.	.75	.85
2. When I receive conflicting information online, I am confident that I can distinguish misinformation from correct information.	.83	.87
3. I am confident that I can contextualize and evaluate the true background and intentions of information on the Internet.	.70	.83
KMO	.704	.704
Bartlett's Test	<.001	<.001

Note. Only one component was extracted. The solution could therefore not be rotated.

Supplementary Table C (continued)

Items	PAF<br>Factor loadings	PCA<br>Factor loadings
Mistrust in media coverage		
1. There is less and less emphasis on fact-checking and source-checking in media reports.	.74	.83
2. More and more false information, so-called "fake news," is being disseminated in the media.	.57	.75
3. The quality of media coverage is getting worse and worse.	.78	.85
KMO	.664	.664
Bartlett’s Test	<.001	<.001

Note. Only one component was extracted. The solution could therefore not be rotated.

1.4 Supplementary Table D.

Overview of how many different information sources were used often and very often by how many respondents (n and percentage).

Number of (different) information sources used often (= "5") and very often (= "6")	*n* respondents	Percentage
0	8	2.2
1	46	12.4
2	93	25.1
3	95	25.6
4	62	16.7
5	33	8.9
6	22	5.9
7	9	2.4
8	3	0.8

Note. n = 371. 7-point scale ranging from 0 ("never") to 6 ("very often"). n and percentage refer to participants using the information source type often (5) and very often (6) on the scale. Scale used only verbal end-labels.
